# Plant Bioactive Constituents and Their Potential Benefits in HPV-Positive Oropharyngeal Squamous Cell Carcinoma—A Narrative Review

**DOI:** 10.3390/cimb48060626

**Published:** 2026-06-16

**Authors:** Violeta Popovici, Emma Adriana Ozon, Manuela Apetrei, Rodica Boca, Cerasela Elena Gîrd

**Affiliations:** 1 Center for Mountain Economics, “Costin C. Kiritescu” National Institute of Economic Research (INCE-CEMONT), Romanian Academy, 725700 Vatra-Dornei, Romania; manuela.apetrei@ce-mont.ro; 2 Faculty of Pharmacy, “Carol Davila” University of Medicine and Pharmacy, 020945 Bucharest, Romania; cerasela.gird@umfcd.ro; 3 Clinical Laboratory, Dorna Medical Clinics, 725700 Vatra-Dornei, Romania; rodicaboca@dornamedical.ro

**Keywords:** head and neck squamous cell carcinoma, human papillomavirus, HPV-positive oropharyngeal squamous cell carcinoma, phytochemicals, therapeutic targets, molecular interactions, mechanistic pathways, nanoformulations, clinical trials

## Abstract

Human papillomavirus (HPV) has become a leading cause of oropharyngeal cancers, alongside well-known risk factors such as tobacco and alcohol use. Currently, HPV-positive oropharyngeal squamous cell carcinoma (HPV+ OPSCC) has increased significantly in developed countries, with HPV-16 being the most common high-risk subtype. Clinically, HPV+ OPSCC shows clear differences in prognosis compared to HPV-negative tumors, particularly regarding survival rates and treatment responses. Patients with HPV+ OPSCC tend to have notably better survival outcomes and a more favorable outlook. Strong evidence indicates that HPV-related oropharyngeal cancers represent a distinct epidemiological, clinical, and molecular group, setting them apart from non-HPV-related cancers. As a result, treatment strategies for these subtypes should follow specific clinical protocols to optimize outcomes. Additionally, the viral oncoproteins E6 and E7, which systematically disrupt host tumor-suppressor networks, provide strong reasons for targeted phytotherapeutic interventions. Therefore, there is increasing interest in exploring plant bioactive compounds with promising anti-HPV and anticancer effects that target key oncogenic pathways. This review aims to compile the latest data on bioactive phytochemicals with mechanistic evidence in HPV+ OPSCC, highlight their molecular interactions across oncogenic signaling pathways, and discuss evidence-based findings focusing on research published from 2000 to 2025.

## 1. Introduction

Head and Neck Squamous Cell Carcinoma (HNSCC) is the seventh most common cancer worldwide, with about 890,000 new cases and 450,000 deaths each year, according to GLOBOCAN estimates. It makes up roughly 4.5% of all cancer diagnoses and deaths [1]. Although traditionally linked to tobacco and alcohol use, recent findings have shed new light on these cancers, especially with the recognition that high-risk human papillomavirus (HPV) is involved [2]. HPV-positive tumors account for 5–20% of all HNSCCs and 40–90% of those arising in the oropharynx, with widely variable rates depending on geographic area, population, and detection assay [3].

In high-income countries, HPV now accounts for 60–80% of oropharyngeal SCC cases [4]. HPV genotype 16 (HPV-16) is the main cause of a growing subset of HPV-HNSCC [5]. It displays a distinct molecular profile and unique epidemiological patterns [6], mainly affecting younger, non-smoking patients, and typically results in a better prognosis than HPV-negative cases [7] (Table 1).

The oncogenic mechanisms of high-risk HPV mainly involve two early viral proteins, E6 and E7, which effectively disrupt the host cell cycle control and apoptosis pathways E6 binds to the tumor suppressor p53 through the cellular ubiquitin ligase E6-AP, leading to its degradation, while E7 inactivates the retinoblastoma protein (pRb) [18,19]. This, in turn, activates E2F transcription factors and promotes uncontrolled cell cycle progression [20]. These changes activate several downstream oncogenic pathways—including NF-κB, PI3K/AKT/mTOR, Wnt/β-catenin, MAPK/ERK, and JAK/STAT—that collectively support cancer cell growth, resistance to cell death, new blood vessel formation, and immune system evasion [21].

HPV-positive oropharyngeal SCC remains a significant challenge in diagnosis and treatment, underscoring the urgent need for innovative strategies [22,23]. Despite significant advances in surgical techniques, radiotherapy, and immunotherapy, especially with agents such as pembrolizumab [24] and nivolumab [25] that target PD-1/PD-L1 [26], the outcomes for recurrent or metastatic HPV+ OPSCC remain with 5-year survival rates around 60% across all stages [12,27,28]. Standard treatments can cause considerable side effects, highlighting the need for new solutions [8]. HPV is a firmly established driver in oropharyngeal HNSCC; it is the only site where the virus is transcriptionally active [29]. HPV is IARC-classified as a Group 1 carcinogen and is independently validated by the p16 surrogate [30].

In HNSCCs of the oral cavity (OSCC) [31,32,33] and larynx (LSCC) [34,35], HPV DNA detection frequently reflects a bystander state rather than active oncogenesis, as E6/E7 mRNA is often absent even in HPV-DNA-positive specimens.

The HPV-HNSCC prognosis depends on the site. HPV positivity confers the most significant survival benefit in OPSCC (>80% disease-free survival rate (DFS) vs. <50% in HPV-negative), is neutral to adverse in OSCC, and is statistically non-significant in LSCC [36]. This gradient directly mirrors the gradient of causal certainty—where HPV is not actively driving carcinogenesis, it logically cannot drive a favorable biology.

Clinical management varies accordingly. Only OPSCC requires mandatory HPV testing, has its own AJCC 8th edition staging, and is the focus of active treatment de-escalation trials [37]. For OSCC and LSCC, HPV status currently does not play a role in guiding staging, treatment intensity, or follow-up protocols—reflecting the scientific consensus that HPV’s biological impact on these sites is insufficient to warrant clinical action. All differences between HPV-positive OPSCC, OSCC, and LSCC are thoroughly illustrated in Table S1 in the Supplementary Material.

In this context, plant-derived compounds have emerged as promising multi-target agents that could effectively address various aspects of HPV-driven cancer development while reducing toxic effects compared to traditional chemotherapies [38,39,40]. This review provides an insightful look at bioactive phytochemicals with proven efficacy against HPV-positive oral cancer. It discusses their molecular targets, interactions with signaling pathways, supporting evidence, and potential clinical applications, highlighting the potential to incorporate these natural products into future treatment approaches. 

## 2. Methods

The limitations of current therapies for HPV+ OPSCC and the need for agents that target HPV-specific oncogenic pathways have led the authors to review recent scientific literature on plant bioactive compounds. Consequently, a comprehensive search was performed using PubMed, Scopus, Web of Science, and Google Scholar for articles published from 2000 to 2025. The search included keywords related to HPV+ OPSCC, HPV E6/E7 oncoproteins, plant bioactive compounds, specific molecular pathways, in vitro evidence in HPV+ OPSCC cell lines, in vivo evidence in animal models, and clinical trials concerning HPV+ OPSCC prevention and posttreatment outcomes. Only peer-reviewed articles written in English and available as full texts in various journals were included. Conference abstracts, theses, dissertations, non-peer-reviewed papers, and studies in other languages were excluded. The selection of phytochemicals focused on multi-target activity and favorable safety profiles, with an emphasis on preclinical and clinical findings.

## 3. Molecular Oncogenesis and Conventional Therapeutic Protocol of HPV+ OPSCC

**Figure 1:**
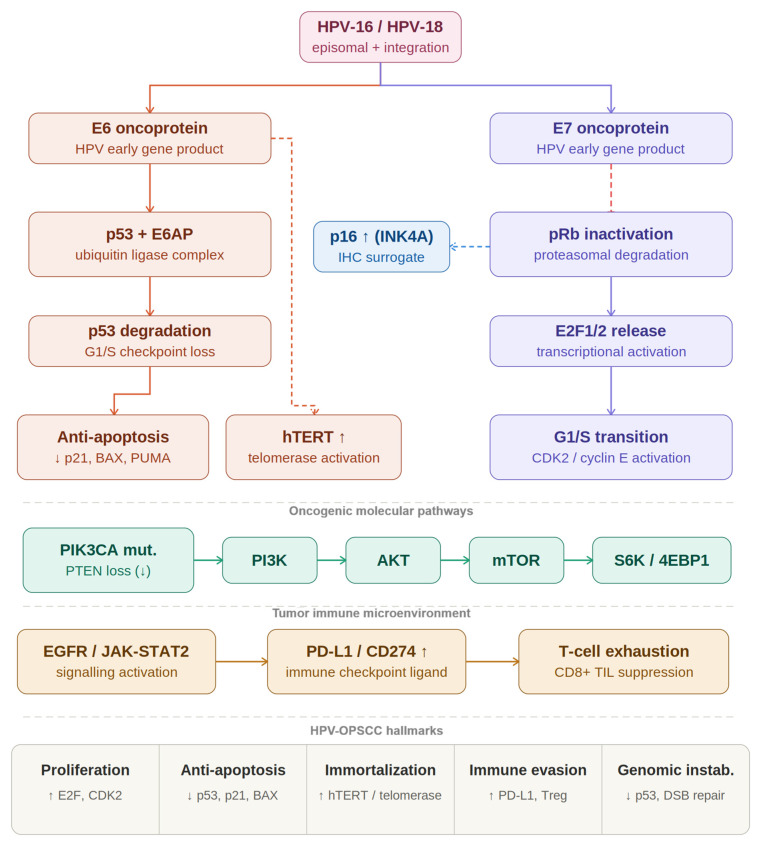
HPV-induced oropharyngeal carcinogenesis. E6 recruits E6AP (a ubiquitin E3 ligase) to form a complex that ubiquitinates p53, targeting it for proteasomal degradation. Loss of p53 abolishes the G1/S DNA-damage checkpoint, suppresses p21/BAX/PUMA-mediated apoptosis, and enables survival under replication stress. E6 also independently activates hTERT, bypassing replicative senescence [49]. E7 binds and degrades pRb (retinoblastoma protein), liberating E2F1/2 transcription factors that drive S-phase gene expression and CDK2/cyclin E activation—forcing cells through the G1→S restriction point. As compensatory feedback, loss of Rb repression leads to accumulation of p16^INK4A (INK4A); this is the basis for p16 immunohistochemistry as a clinical HPV surrogate marker. PIK3CA gain-of-function mutations and PTEN loss (the most common co-operating alteration in HPV+ OPSCC) activate the PI3K → AKT → mTOR → S6K/4EBP1 axis, promoting cap-dependent translation, cell survival, and metabolic reprogramming. EGFR and JAK-STAT2 signaling upregulate PD-L1/CD274 on tumor cells. Despite the characteristically high CD8+ TIL infiltration in HPV+ OPSCC (which partly explains the better prognosis), PD-L1 engagement induces T-cell exhaustion—the rationale for anti-PD-1/PD-L1 checkpoint inhibition (pembrolizumab) in recurrent/metastatic disease. All pathways converge on uncontrolled proliferation, resistance to apoptosis, replicative immortality, immune evasion, and genomic instability due to deficient p53-mediated DNA repair.

The molecular oncogenesis of HPV-positive oropharyngeal cancer is a multi-step process initiated by persistent HPV-16 infection [41]. The viral E6 and E7 oncoproteins [42] disrupt critical tumor suppressors (p53 and Rb), while simultaneously activating inflammatory pathways, particularly the IL-6/STAT3 axis [43,44,45]. Additional somatic mutations (NOTCH1, SOX2) and the presence of extrachromosomal DNA further drive malignant transformation [46]. The tumor microenvironment, including immune cell populations and hypoxic conditions, plays a crucial modulatory role in disease progression and treatment response [47,48]. This complex interplay of viral, genetic, and microenvironmental factors distinguishes HPV+ OPSCC from other head and neck cancers. It explains its unique clinical characteristics, including improved initial prognosis but heterogeneous treatment responses (Figure 1).

### 3.1. The E6/p53 Axis

HPV16-E6, approximately 150 amino acids long, contains two zinc-binding domains that create a surface for binding p53. When E6, E6-AP (an E3 ubiquitin ligase), and p53 form a ternary complex, p53 is rapidly ubiquitylated and degraded by the proteasome—a process that has recently been understood at atomic detail [50]. This has significant consequences: loss of p53-controlled G1/S and G2/M checkpoints, weakened DNA damage response, reduced apoptosis due to lower BAX levels, and less p21 (CDKN1A) induction [51,52,53,54,55,56,57]. Additionally, E6 interferes with the p53-p300-CBP transcriptional activator complex, decreasing p53 acetylation and limiting its activity even when protein levels remain relatively stable [58]. From a phytotherapeutic perspective, the E6/p53 pathway is a particularly promising target [59,60,61]. Compounds that can inhibit E6 gene transcription, destabilize E6 protein, or competitively bind to E6 interaction sites could, in theory, restore proper p53 signaling and reactivate the cell’s innate apoptotic pathways—similar to how MDM2 inhibitors have been effectively used in HPV-negative p53 wild-type cancers [62,63].

### 3.2. The E7/pRb Axis and Cell-Cycle Dysregulation

The HPV16-E7 protein binds to pRb via a conserved LXCXE motif, preventing pRb from sequestering E2F transcription factors [64]. Consequently, E2F1-3 can activate genes essential for S-phase entry, such as Cyclin E, Cyclin A, and CDK2 [65]. Furthermore, E7 encourages the ubiquitination and proteasomal degradation of pRb [64,66]. A recently discovered additional mechanism involves E7 disrupting the DREAM complex [67], a repressive complex involving E2F4/5, leading to widespread activation of cell-cycle genes independent of E6′s influence on p53. The overexpression of p16INK4A—used clinically as a surrogate marker for HPV—serves as a compensatory feedback loop within the pRb pathway, rather than indicating effective tumor suppression [68].

### 3.3. Downstream Oncogenic Signaling Networks

The loss of p53 and pRb function triggers multiple oncogenic pathways that are further enhanced in HPV-positive OHSCC:

○ The NF-κB pathway, activated by viral proteins and inflammatory stimuli, promotes transcription of anti-apoptotic genes (Bcl-2, cIAP-2, Bcl-xL), pro-angiogenic factors (VEGF, IL-8), and invasion-related genes (MMP-2, MMP-9, E-selectin) [69,70,71].
○ The PI3K/AKT/mTOR pathway is often activated through EGFR overexpression (seen in about 90% of OPSCC), PTEN loss, or direct viral influence; it promotes proliferation, metabolic reprogramming, and resistance to apoptosis [72,73,74,75]
○ The Wnt/β-catenin pathway supports cancer stem cell maintenance, epithelial–mesenchymal transition (EMT), and resistance to treatment; nuclear β-catenin accumulation has been observed in HPV-positive OPSCC [76].
○ In the JAK/STAT pathway, STAT3 is constantly active in OPSCC, aiding immune evasion by upregulating PD-L1 and inhibiting dendritic cell maturation [77,78,79].
○ The MAPK/ERK pathway, downstream of EGFR and RAS, promotes cell proliferation and survival; RAS mutations that activate this pathway are less common in HPV-positive than in HPV-negative OPSCC [80,81,82].

### 3.4. Immune Evasion in HPV+ OPSCC

Despite the immunogenic nature of HPV infection, tumors effectively evade immune surveillance through multiple mechanisms: E6-mediated repression of E-cadherin disrupts antigen presentation; E7 promotes immune exclusion via MARCHF8-dependent degradation of FAS and TRAIL death receptors; HIF-1α-driven PD-L1 upregulation inactivates tumor-infiltrating T cells; and myeloid-derived suppressor cells (MDSCs) and regulatory T cells (Tregs) accumulate in the tumor microenvironment (TME) [83,84,85,86].

### 3.5. Conventional Therapeutic Protocol for HPV+ OPSCC

**Figure 2:**
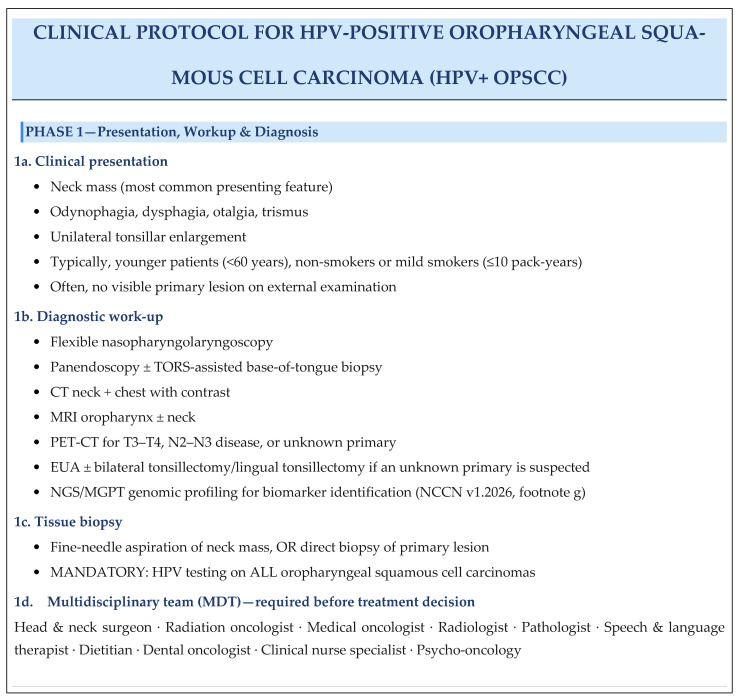

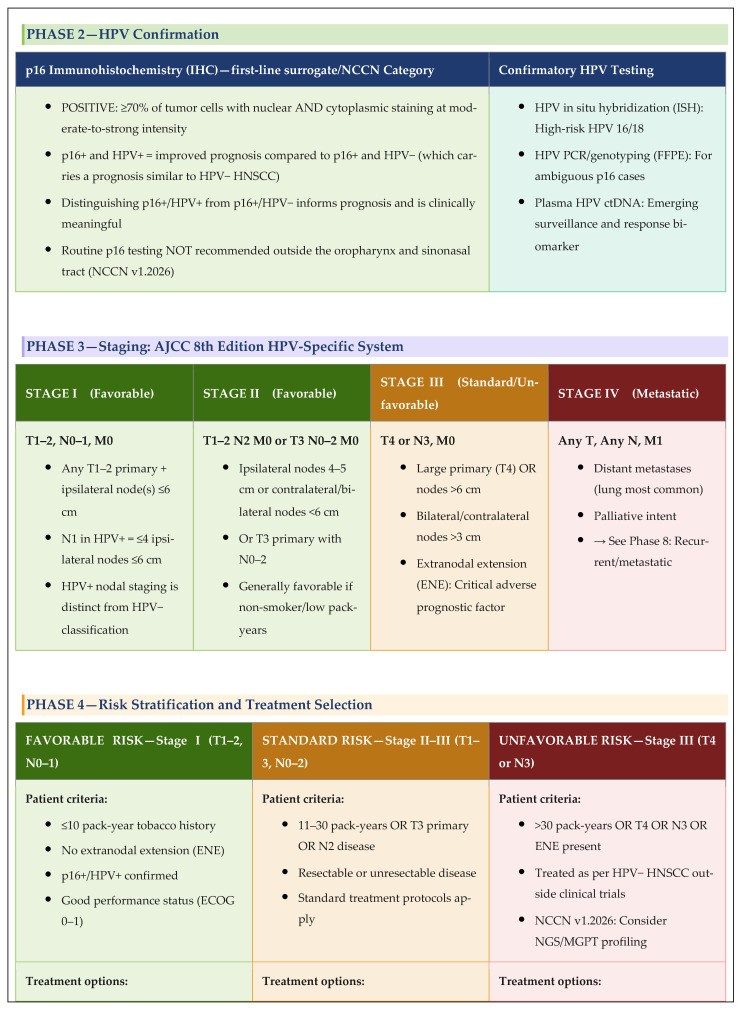

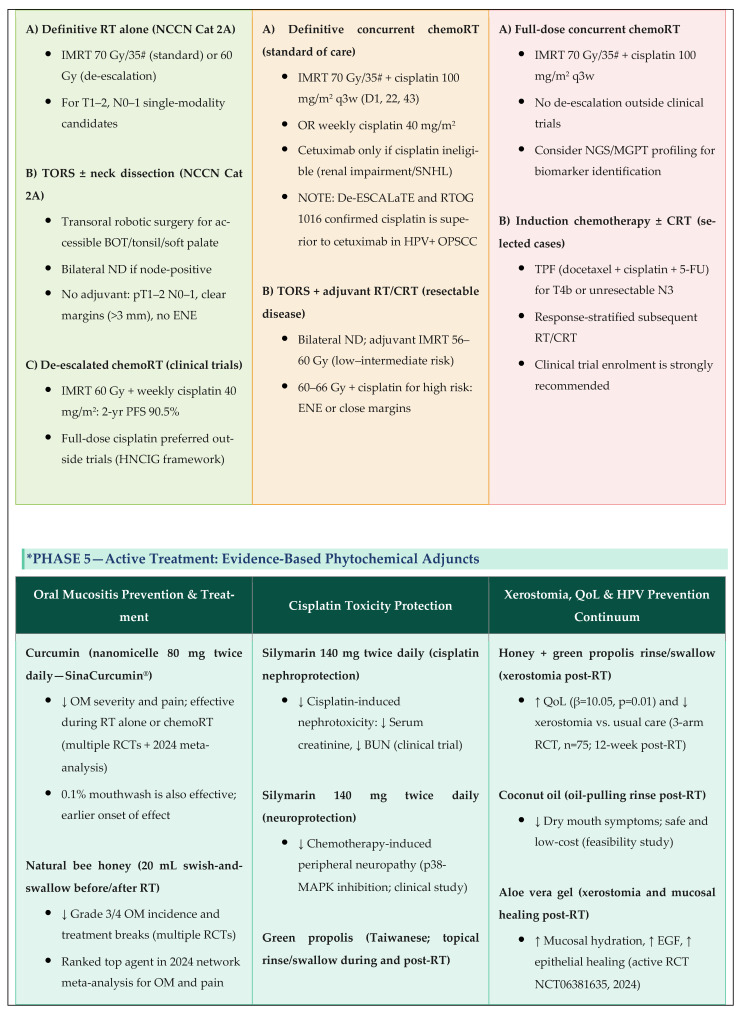

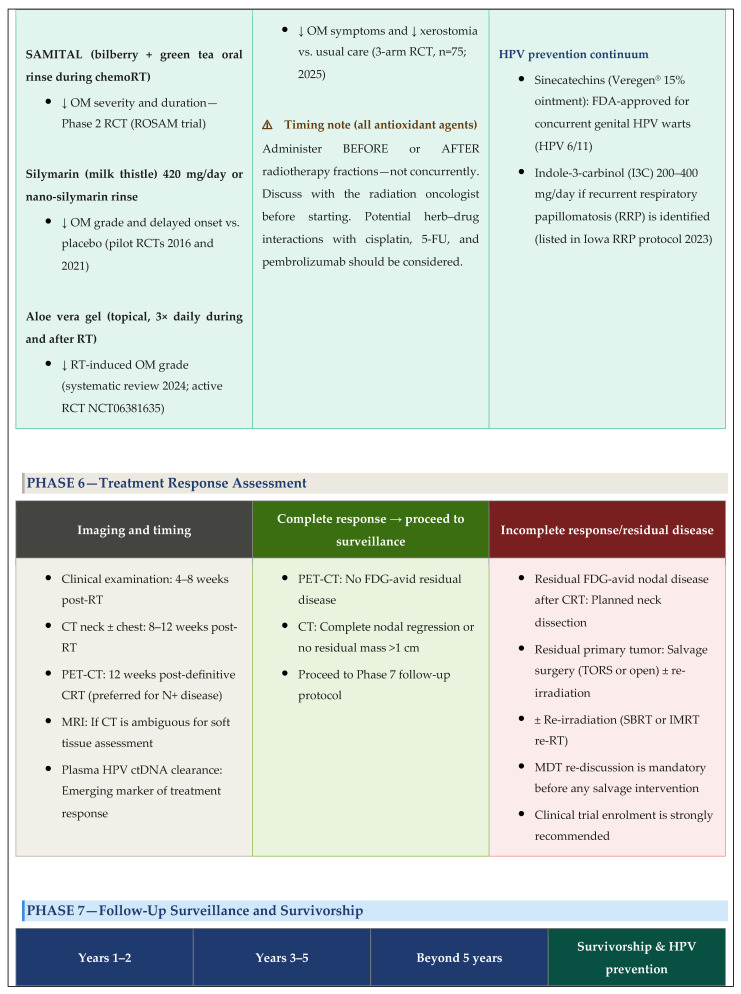

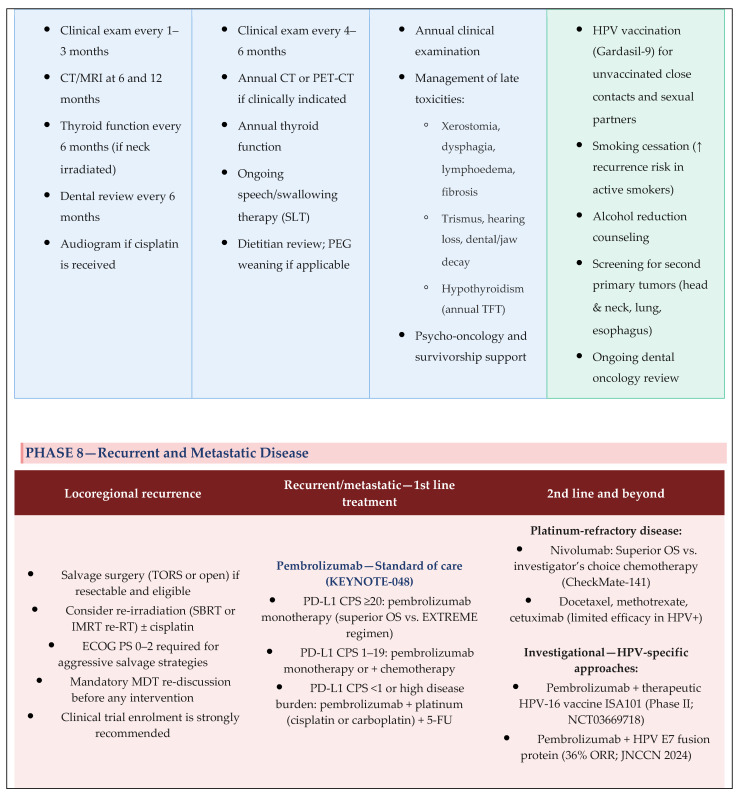
Clinical protocol for HPV+ OPSCC. * Phase 5 includes the evidence-based findings of the present study. IHC—INK4a immunohistochemistry; p16 IHC ≥70% strong diffuse staining—this result indicates a strongly positive test, showing the presence of a transcriptionally active High-Risk Human Papillomavirus (HR-HPV) infection, according to Ablaza AC, Gupta R, Fielder T. p16. PathologyOutlines.com website, at https://www.pathologyoutlines.com/topic/stainsp16.html, accessed on 30 May 2026; 8th Edition of AJCC—American Joint Committee on Cancer [87]; TNM system, according to https://www.cancer.gov/about-cancer/diagnosis-staging/staging, accessed on 31 May 2026; T—the main tumor size and extension; N—the number of nearby lymph nodes with cancer; M—metastases; Database for Phases 1, 2, 4, 7, and 8 was National Comprehensive Cancer Network. Head and Neck Cancers, NCCN Guidelines Version 1.2026, available online at https://www.nccn.org/, accessed on 30 May 2026ș 5-year OS—5-year overall survival rate; CRT—chemoradiation; ECE—extracapsular extension; ECOG PS—Eastern Cooperative Oncology Group performance status; IHC—immunohistochemistry; IMRT—intensity-modulated radiotherapy; Gy—Gray; Fr—Fraction; ISH—in situ hybridization; LVI—lymphovascular invasion; MDT—multidisciplinary team; ND—neck dissection; PNI—perineural invasion; R/M—recurrent/metastatic; RT—radiotherapy; SND—selective neck dissection; TORS—transoral robotic surgery; q3w—every three weeks; TPF—docetaxel + cisplatin + 5-fluorouracil; 5-FU—5-fluorouracil; ORN—osteoradionecrosis.

The conventional therapeutic protocol for HPV-positive oral cancer is shown in Figure 2. The figure outlines the standard-of-care pathway for HPV-positive OPSCC using the AJCC 8th edition staging system, which specifically reclassified p16+/HPV+ tumors as a separate, more favorable category The improved prognosis of HPV+ OPSCC compared to HPV-negative oral cancers has driven extensive research into treatment de-escalation to reduce long-term toxicity without affecting oncological outcomes.

#### 3.5.1. Low-Risk Status (Stage I–II)

These patients are candidates for de-escalation strategies to reduce treatment intensity, such as less invasive surgery, lower radiation doses, or reduced chemotherapy [88,89,90]. This approach decreases long-term side effects and improves patients’ quality of life without compromising high survival rates [91]. When surgical access allows, transoral robotic surgery (TORS) or transoral laser microsurgery (TLM) is preferred [92,93,94], with reduced-dose adjuvant therapy if pathological risk factors like positive margins or extracapsular extension are absent. Radiation alone at a lower dose (60–66 Gy) is an alternative [95,96]. Several clinical trials (e.g., ECOG-ACRIN 3311, PATHOS) are actively refining these thresholds [93,95,96,97,98] (Figure 2).

#### 3.5.2. Locally Advanced (Stage III–IV)

Concurrent chemoradiation remains the standard, typically IMRT to 70 Gy with cisplatin (100 mg/m^2^ q3w) [93]. Post-CRT PET-CT at 12 weeks guides the decision for salvage neck dissection [99,100]. Surveillance is intensive during the first two years [101,102,103,104,105,106], then tapers off, with focus on thyroid function (if the neck was irradiated) [107], swallowing therapy [108,109,110,111,112,113,114,115] and smoking cessation (since smoking worsens prognosis even in HPV+ disease) [116,117,118,119,120]. (Figure 2).

## 4. Plant Bioactive Constituents: Mechanistic Evidence

**Figure 3:**
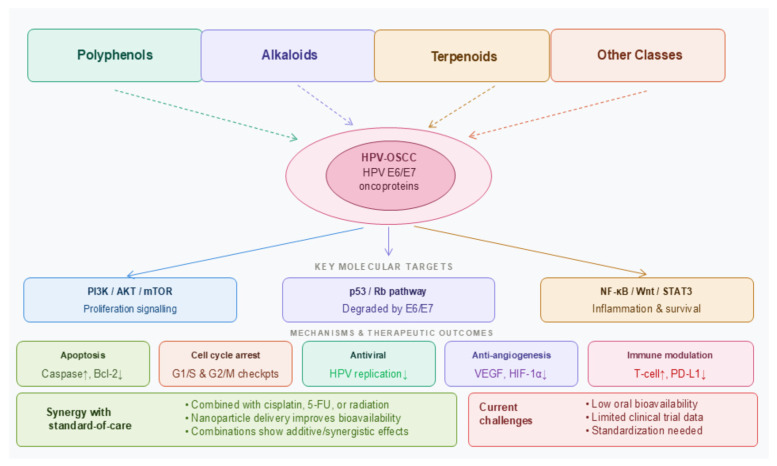
Plant bioactive constituents with potential benefits in HPV-OPSC.

Four major categories of plant bioactive compounds were selected: polyphenols, alkaloids, terpenoids, and other classes (Figure 3).

### 4.1. Curcumin

Curcumin, (1*E*,6*E*)-1,7-bis(4-hydroxy-3-methoxyphenyl) hepta-1,6-diene-3,5-dione, is the principal bioactive polyphenol extracted from the rhizome of turmeric (*Curcuma longa*), a member of the Zingiberaceae family. It belongs to the curcuminoid class and has been investigated in over 150 human clinical trials across oncological and inflammatory conditions.

Mishra et al. [121] demonstrated that curcumin is a powerful and highly selective inhibitor of HPV16-E6 oncogene transcription in the HPV16-positive oral carcinoma cell line 93VU147T. It works by blocking the transcriptional activators AP-1 and NF-κB, which normally bind to the HPV upstream regulatory region (URR) to promote E6 and E7 gene expression. Immunoblot analyses show an encouraging inverse relationship: as curcumin levels increase, E6 protein decreases, and p53 protein is restored [122]. At the same time, the levels of anti-apoptotic proteins Bcl-2 and cIAP-2 decline, while pro-apoptotic BAX increases—highlighting the reactivation of p53-dependent apoptosis [123].

Other authors offered a thorough review of curcumin’s mechanisms of action across multiple pathways in head and neck cancer [124]. They confirm that its cell-killing effects in HPV+ cell lines are mainly due to E6 suppression and the subsequent restoration of p53. The review covers curcumin’s effects on:

○ Wnt/β-catenin: It inhibits Wnt ligand–receptor interaction and promotes GSK3β-mediated phosphorylation and degradation of β-catenin, which helps reduce cancer stem cell renewal [125].
○ PI3K/AKT/mTOR: It suppresses PI3K activity and AKT phosphorylation. This downregulation decreases mTOR activity, which in turn reduces the translation of oncoproteins, as shown in oral cancer cells by blocking EGFR phosphorylation and ERK1/2 [126].
○ JAK/STAT3: Curcumin reduces STAT3 phosphorylation at Y705, leading to lower transcription of genes like Cyclin D1, c-Myc, and PD-L1 [127].
○ Notch signaling: It decreases Notch-1 and its downstream targets HES1/HEY1, thereby reducing invasion and EMT in OSCC cell lines [124].
○ miRNA-31 regulation: Curcumin lowers the levels of miR-31 (an oncomiR that is overexpressed in OSCC), which reduces AKT activation and C/EBPβ-driven transcription [128].
○ Epigenetic reprogramming: It inhibits DNMT1 and HDAC activities, reactivating silenced tumor suppressor genes [129].
○ Ferroptosis induction: Recent research has identified this as an additional mechanism, involving GPX4 modulation and lipid peroxide accumulation, that contributes to cancer cell death [130].

It was also reported that copper supplementation enhances curcumin’s cytotoxicity in oral cancer cell lines [131].

### 4.2. (+/−)-Epigallocatechin Gallate

(+/−)-Epigallocatechin Gallate-13C3 (EGCG, [2*R*,3*R*)-5,7-dihydroxy-2-(3,4,5-trihydroxyphenyl)-3,4-dihydro-2*H*-chromen-3-yl] 3,4,5-trihydroxybenzoate) is the most abundant and biologically active catechin in green tea (*Camellia sinensis*, Theaceae). It makes up about 40–60% of the total catechin content in fresh tea leaves and is classified as a flavan-3-ol polyphenol [132].

Tsouh et al. demonstrated that EGCG: (1) helps slow the growth of HNSCC cell lines and promotes cell death; (2) decreases pEGFR, a key factor in promoting cancer in OSCC; (3) lowers Cox-2 levels, which helps reduce immunosuppression caused by prostaglandin E2; (4) suppresses activated STAT3 (pSTAT3), leading to less PD-L1 production; and (5) reduces Cyclin D1, supporting G1 cell-cycle arrest [133]. EGCG also binds to the proteins Bcl-2 and Bcl-xL, preventing their inhibition of cell death and thus promoting apoptosis. Furthermore, EGCG can disrupt lipid rafts in OPSCC cells, displacing EGFR from its signaling hubs and reducing downstream cancer-promoting signals [134].

EGCG targets HPV at multiple points: It blocks the virus from entering cells by binding to HPV L1 capsid protein, preventing attachment to epithelial cells—beneficial for both prevention and treatment. It also lowers E6 and E7 levels by inhibiting NF-κB and AP-1, reducing their gene activity [135,136]. Additionally, recent research shows that EGCG can inhibit PD-L1 expression by suppressing NF-κB, helping to restore CD8+ T cell activity in tumors—an important finding for HPV-positive OPSCC, where PD-L1/PD-1 inhibitors are already approved treatments [137,138]. Recent reviews confirm that EGCG has anticancer activity, especially against HPV16-related cancers, and that this promising effect is dose-dependent [139,140].

Recently, a standardized green tea extract called Polyphenon E, marketed as Veregen^®^, was FDA-approved for treating external genital warts caused by HPV [141].

### 4.3. Resveratrol

Resveratrol, 5-[*E*)-2-(4-hydroxyphenyl) ethenyl] benzene-1,3-diol, is a naturally occurring compound classified as a stilbene polyphenol. It is produced as a defensive response, called a phytoalexin, mainly in grapes (*Vitis vinifera*), blueberries, mulberries, peanuts, and most abundantly in *Polygonum cuspidatum*. This compound has two isomeric forms, cis and trans, with the “trans” form being biologically active.

Resveratrol can decrease HPV E6 and E7 mRNA and protein levels in HPV16-positive (CaSki) and HPV18-positive (HeLa) cervical cancer cells [142]. These findings could be relevant for HPV-positive OPSCC because they involve similar viral cancer mechanisms. The proposed idea was that resveratrol interrupts the splicing of the E6 intron from the E6E7 pre-mRNA, a crucial step after transcription. This leads to several benefits: (1) restoring the expression of tumor suppressors p53 and p16INK4A; (2) raising BAX levels and lowering Bcl-2, thereby encouraging apoptosis; (3) causing G1/S cell cycle arrest through p21 activation; and (4) reducing phospho-pRb1, counteracting the effects of E7 on cell cycle control [142].

Some studies expanded on these findings by showing that resveratrol and its more bioavailable form, pterostilbene, also decrease HPV E6 levels and inhibit NF-κB activity in HPV-positive cancer cells [143,144]. Researchers also found that combining resveratrol with other polyphenols produces a synergistic effect, leading to the development of TriCurin [145]. In addition to targeting viral proteins, resveratrol helps shut down persistent NF-κB activation in head and neck cancers, reducing the secretion of inflammatory factors such as TNF-α, IL-6, and VEGF [146,147]. It also inhibits STAT3 phosphorylation and lowers MMP-2 and MMP-9 levels, helping to prevent cancer invasion [147]. Its ability to stop metastasis is partly due to its suppression of EMT, as indicated by lower levels of vimentin, E-cadherin, and Snail [148].

### 4.4. TriCurin: A Synergistic Polyphenol Combination

TriCurin is a rationally designed combination of three food-derived polyphenols: curcumin (from *Curcuma longa*), epicatechin gallate (ECG, from green tea), and resveratrol (from grapes), combined at the specific molar ratio of 4:1:12.5 [149].

A detailed analysis of various two- and three-way combinations of polyphenols against HPV-positive cervical cancer cells (HeLa) and HPV16+ head and neck cancer cells (TC-1) [145,150]. Using combination index (CI) analysis, they identified a particularly effective mix, TriCurin. This combination showed the strongest synergy (CI < 1). In HPV16+ TC-1 cells, which produce c-Ha-ras and HPV16-E6/E7, TriCurin significantly increased HPV E6 protein suppression by 4.7 times, doubled p53 induction, raised activated p53 (acetyl-p53) sixfold, and elevated activated caspase-3 levels by 1.7 times compared to curcumin alone [151]. When tested in live animals, intralesional injections of TriCurin into subcutaneous TC-1 tumors in C57BL/6 mice resulted in an impressive 80–90% reduction in tumor growth during treatment, all without harmful effects on healthy control mice. The treatment works by the three compounds acting together to interfere with the E6–p300/CBP interaction: curcumin and resveratrol first reduce E6 transcription by suppressing NF-κB and AP-1 pathways. At the same time, ECG enhances p300 acetyl transferase activity, shifting the balance toward p53 acetylation and activation [58]. Piao et al. further explored TriCurin’s effects specifically on HPV-positive HNSCC, confirming that it can effectively target HPV+ HNSCC cell lines without harming normal keratinocytes—highlighting its promising potential as a safe and effective treatment option in the oropharyngeal area [151].

### 4.5. Quercetin

Quercetin, 2-(3,4-dihydroxyphenyl)-3,5,7-trihydroxychromen-4-one, is common in plants and is found in large amounts in onions (*Allium cepa*), elderberries, capers, kale, and tea. It is considered the most widely consumed dietary flavonoid worldwide.

Quercetin’s anticancer effects on various OSCC cell lines were investigated using various methods (MTT assay, flow cytometry, wound-healing assay, invasion assay, and gelatin zymography). Key findings include: (1) inducing a selective G2/M cell-cycle arrest—by suppressing Cyclin B1/CDK1—without affecting normal human oral keratinocytes (HaCaT, nHOK); (2) blocking TGF-β1-induced EMT by targeting the Slug transcription factor, which decreases fibronectin and vimentin levels; (3) reducing MMP-2 and MMP-9 activity, thereby limiting basement membrane invasion [152,153,154,155,156,157].

Molecular docking study confirmed quercetin’s strong binding affinity for the Bcl-2 protein, outperforming the reference ligand doxorubicin, and provided a structural basis for its pro-apoptotic activity [158]. Other studies reaffirmed its effects on cancer invasion, migration, proliferation, and apoptosis induction across multiple OSCC model systems [158,159,160,161].

Regarding HPV-specific activity, quercetin suppresses NF-κB activity, similar to curcumin and EGCG. In vitro studies of HPV+ cancer cells show decreased E-cadherin repression and increased T-cell-mediated killing compared to untreated HPV+ controls, indicating potential indirect immune-boosting effects in the HPV-positive environment. Moreover, the combination of quercetin and cisplatin increases apoptosis of OSCC cells by downregulating XIAP through the NF-kB pathway [162]. Quercetin also enhances the sensitivity of oral cancer cells to Vincristine [163].

### 4.6. Berberine

Berberine, 16,17-dimethoxy-5,7-dioxa-13-azoniapentacyclo [11.8.0.02,10.04,8.015,20]henicosa-1(13),2,4(8),9,14,16,18,20-octaene, is an isoquinoline alkaloid found in *Berberis vulgaris* (barberry), *Berberis aristata*, *Hydrastis canadensis* (goldenseal), and *Coptis chinensis* (Chinese goldthread). It has a long history of use in traditional Chinese and Ayurvedic medicine [164].

Berberine has dual actions—tumor growth inhibition and immune restoration—especially relevant in OPSCC, where both the virus and the tumor cause immunosuppression. Berberine specifically: (1) disrupts the IL-6/STAT3 axis in cancer-associated fibroblasts, lowering VEGF and PD-L1 levels; (2) stabilizes endothelial tight junctions and reduces oxidative damage, normalizing tumor blood vessels and enhancing T-cell infiltration; (3) reduces immunosuppressive MDSCs in the tumor environment, allowing more effective activation of cytotoxic T-cells; (4) destabilizes PD-L1 protein via STUB1-mediated ubiquitylation, decreasing immune checkpoint engagement [165,166].

Berberine also suppresses telomerase activity in cancer cells—a mechanism especially important in HPV-positive cancers, where E6 increases hTERT (human telomerase reverse transcriptase), leading to cellular immortalization [167,168].

These plant metabolites act through various mechanisms in HPV+ OPSCC cell lines (Table 2).

### 4.7. Apigenin

Apigenin (5,7-dihydroxy-2-(4-hydroxyphenyl)chromen-4-one) is a flavone subclass flavonoid found in high amounts in *Petroselinum crispum* and *Matricaria chamomilla*. It has attracted researchers’ attention for its ability to slow cell growth and its gentle effect on normal cells.

A recent study examined curcumin, EGCG, and apigenin together in the context of HPV-related cancer [134]. They noted that apigenin interacts with the PI3K/AKT, NF-κB, MAPK/ERK, and Wnt/β-catenin pathways, which are often disrupted in HPV-positive OSCC [134]. Its anti-HPV effects are more indirect, by (1) reducing NF-κB activity, which leads to less E6/E7 expression driven by HPV URR, (2) lowering STAT3 phosphorylation, which decreases PD-L1 and helps the immune system, (3) cutting down Cox-2 and prostaglandin E2, which helps reduce inflammation that can promote tumors, (4) directly encouraging cancer cell death through caspase-3/7 activation and PARP cleavage in OSCC cells and (5) working together with EGCG to boost cancer cell death even more, with the combination showing stronger effects at doses that are safe on their own [134,178].

### 4.8. Luteolin

Luteolin, 2-(3,4-dihydroxyphenyl)-5,7-dihydroxychromen-4-one, is commonly found in plants from the Asteraceae, Lamiaceae, Poaceae, Leguminosae, and Scrophulariaceae families. It has been the most extensively studied flavonoid for directly inhibiting HPV oncoproteins. Luteolin showed a significant, dose-dependent cytotoxic effect only in HPV-positive cancer cells, not in HPV-negative cells. It suppressed the expression of HPV E6 and E7 oncogenes, restored levels of pRb and p53, and increased E2F5 levels. Additionally, luteolin enhanced the expression of death receptors (Fas/FasL, DR5/TRAIL, FADD), activated caspase cascades—specifically caspase-3 and -8—induced mitochondrial membrane potential collapse, caused cytochrome c release, and inhibited Bcl-2 and Bcl-xL [179,180,181].

Using structure-based virtual screening of the HPV-16 E6–E6AP interface, luteolin emerged as the most potent inhibitor of the E6–E6AP interaction, which is essential for p53 degradation—providing a direct mechanistic rationale for its HPV-selective activity [182].

### 4.9. Indole-3-Carbinol

Indole-3-carbinol (I3C, 1H-indol-3-ylmethanol) is a product of glucosinolate hydrolysis found in cruciferous vegetables (such as broccoli, cabbage, cauliflower, kale, and mustard) [178]. In the acidic environment of the stomach, I3C further condenses to form diindolylmethane (DIM), which contributes to many of I3C’s bioactivities [183,184].

I3C-specific anti-HPV mechanisms consist of the following: (1) I3C counteracts HPV16 immune evasion by antagonizing E6 protein-mediated repression of E-cadherin, restoring cell-surface adhesion molecule expression and enhancing antigen presentation to cytotoxic T lymphocytes; (2) I3C functions as a class I HDAC inhibitor, derepressing tumor suppressor genes silenced by E7-driven epigenetic modifications; (3) I3C decreases proinflammatory cytokines (TNF-α, IL-1β, IL-6) and T-cell activation by disrupting HDAC-dependent transcriptional programs [185].

Indole-3-carbinol inhibits nasopharyngeal carcinoma cell growth in vivo and in vitro through apoptosis based on the PI3K/Akt pathway [186].

### 4.10. α-Mangostin

α-Mangostin (α-MG, 1,3,6-trihydroxy-7-methoxy-2,8-bis(3-methylbut-2-enyl) xanthen-9-one) is a xanthone derivative extracted from the pericarp (peel) of the tropical mangosteen fruit (*Garcinia mangostana*, Clusiaceae). It is a polyhydroxylated compound with strong antioxidant, anti-inflammatory, and antineoplastic effects.

Díaz et al. [187] provided direct molecular evidence that α-Mangostin inhibits cervical cancer cell proliferation and tumor growth by downregulating the E6/E7 HPV oncogenes and the KCNH1 potassium channel gene, an E6/E7-regulated driver of proliferation. The inhibitory effects were proportional to the HPV copy number—the cell line with the highest HPV load (CaSki with HPV16) showed the greatest sensitivity—directly implicating the E6/E7 suppression mechanism [187].

The anti-OSCC evidence for α-MG, highlights the following mechanistic pathways: (1) G1-phase cell cycle arrest through downregulation of CDK4/CDK6 and Cyclin D1; (2) activation of the intrinsic (mitochondrial) apoptosis pathway via collapse of mitochondrial membrane potential, cytochrome c release, and caspase-9/caspase-3 activation; upregulation of pro-apoptotic BAX and downregulation of anti-apoptotic Bcl-2 [188,189].

Critically, a recent clinical-stage development study tested a 1% α-mangostin orabase gel formulation directly against OSCC cells [190]. Additionally, mucoadhesive films loaded with α-MG demonstrated anti-HPV16 activity at both attachment and post-attachment stages, establishing dual antiviral and anticancer activities within a single mucosal delivery format [191].

### 4.11. Silymarin

Silymarin is the standardized extract derived from the seeds of milk thistle (*Silybum marianum, Asteraceae*), comprising approximately 65–80% silibinin, along with silydianin and silychristin. Silibinin is the most pharmacologically active constituent.

Silibinin, (2*R*,3*R*)-3,5,7-trihydroxy-2-[(2*R*,3*R*)-3-(4-hydroxy-3-methoxyphenyl)-2-(hydroxymethyl)-2,3-dihydro-1,4-benzodioxin-6-yl]-2,3-dihydrochromen-4-one, has been shown to inhibit STAT3 and NF-κB signaling pathways in head and neck squamous cell carcinoma (HNSCC) cell lines, thereby decreasing the expression of genes related to survival and inflammation [192]. In the tumor microenvironment of oral squamous cell carcinoma (OSCC), STAT3 activation increases PD-L1 levels and hinders dendritic cell maturation. The inhibition of STAT3 by silybin offers both direct anti-proliferative effects and indirect immunity-enhancing benefits [193,194].

Silymarin was also shown to have anti-HPV activity, as confirmed by molecular docking [195].

Numerous in vitro studies have been conducted on HPV-positive cervical cancer cell lines. HPV-related cervical cancer and HPV+ OPSCC share identical viral drivers and core molecular mechanisms and show excellent treatment responses. Both are caused by high-risk HPV strains (mainly HPV-16) and differ from virus-negative cancers in their unique cellular and prognostic profiles. The mechanistic pathways involved in their cytotoxic activity against HPV-induced cervical cancer are shown in Table 3.

## 5. Convergent Signaling Pathway Analysis

Analysis of molecular targets across all 15 compounds shows clear convergence on a core set of oncogenic nodes that are amplified or derepressed by HPV E6/E7 activity. This convergence has significant implications for the design of combination therapies.

### 5.1. The E6/p53 Restoration Axis

Curcumin, resveratrol, TriCurin, and α-Mangostin all restore p53 function through complementary mechanisms: curcumin and resveratrol suppress HPV URR transcriptional activity; resveratrol also interferes with E6/E7 mRNA splicing; α-Mangostin suppresses E6/E7 at the transcriptional level. TriCurin’s synergy results from simultaneously blocking E6 transcription and increasing p53 acetylation (activation), thereby restoring p53-dependent apoptosis in a feed-forward manner.

### 5.2. NF-κB Pathway Suppression

NF-κB acts as a convergence point for at least eight compounds (curcumin, EGCG, resveratrol, quercetin, apigenin, silymarin, artemisinin, and berberine). In HPV-positive OSCC, persistent NF-κB activation promotes both viral E6/E7 transcription and host oncogenic processes. The variety of compounds targeting this pathway explains why combining polyphenols often produces synergistic effects at doses lower than their individual effective levels.

### 5.3. PI3K/AKT/mTOR Axis

EGFR overexpression in approximately 90% of OPSCC cases activates the PI3K/AKT/mTOR pathway. Curcumin (by inhibiting EGFR and PI3K), EGCG (by disrupting EGFR lipid rafts), quercetin (by targeting BCl-2/BCl-xL), resveratrol (by inhibiting Akt phosphorylation), and apigenin (by suppressing PI3K) all engage this pathway. Importantly, mTOR inhibition by curcumin and resveratrol reduces cap-dependent translation of oncoproteins, including Cyclin D1 and c-Myc, thereby increasing G1 arrest.

### 5.4. Tumor Microenvironment and Immune Remodeling

HPV+ OPSCC has a relatively high tumor mutational burden and an immunologically ‘hot’ microenvironment. Yet, it systematically evades immune killing through PD-L1 upregulation (STAT3-dependent), MDSC accumulation, and loss of death receptor expression. EGCG (PD-L1 suppression via NF-κB), berberine (PD-L1 destabilization; MDSC reduction), curcumin (anti-PD-L1 through STAT3 inhibition; TME reversal), and I3C (E-cadherin restoration; HDAC inhibition) form a multi-compound immunological toolkit that could effectively improve the efficacy of anti-PD-1 checkpoint inhibitors (Table 4).

## 6. Bioavailability Challenges and Nanoscale Delivery Systems 

The main obstacle to using phytochemicals in clinical settings is their poor pharmacokinetics: most polyphenols have low water solubility, undergo extensive first-pass metabolism (glucuronidation, sulphation), are quickly cleared from the body, and therefore reach low plasma levels after oral intake. For example, the oral bioavailability of curcumin in humans is estimated to be less than 1% when administered as a free compound. 

Therefore, nanoscale delivery systems present a valuable approach to overcoming the challenges faced by traditional plant-derived medicines, such as poor water solubility, chemical instability, and low bioavailability. By encapsulating bioactive phytochemicals within microscopic carriers, these advanced systems enhance systemic circulation, improve cellular absorption, and facilitate targeted or controlled release at the site of disease. Thus, nanoparticle-based delivery systems have been shown to significantly improve the therapeutic potential of phytochemicals, making them promising candidates for safer, more effective cancer treatments. The convergence of nanotechnology with phytochemical therapy represents a paradigm shift for HPV+ OPSCC treatment [210]. 

### 6.1. Liposomal Nanovesicles

Liposomes are phospholipid bilayer vesicles capable of encapsulating both hydrophilic and lipophilic phytochemicals, making them highly versatile for polyphenolic compounds that are relevant to HPV-driven oncogenesis. Liposomal nanovesicles exhibit enhanced transport across cell membranes and mucosal barriers, cancer-specific targeting, improved stability of encapsulated phytochemicals against degradation, and controlled, sustained release at tumor sites. 

Given that HPV+ OPSCC tumors are in the oropharynx, liposomal systems can be engineered to exhibit mucoadhesive properties, thereby prolonging contact time with the tumor microenvironment. Polymer-coated mucoadhesive liposomes can remain attached to intestinal/mucosal surfaces for sufficient periods to enable prolonged drug absorption. Furthermore, topical liposomal antiviral delivery has been specifically explored for HPV infections, demonstrating improved drug penetration and localized action. Liposomal nanovesicles offer a compelling and effective strategy for delivering plant bioactive compounds in the fight against HPV+ OPSCC. One of their notable advantages is the ability to enhance the bioavailability of curcumin, resveratrol, and epicatechin gallate [211]. This ensures sustained and concentrated exposure to critical targets, including the HPV16 E6/E7 oncoproteins, the p53/pRB tumor suppressor pathways, and the EGFR/PI3K signaling networks—key components in HPV-driven oropharyngeal carcinogenesis. Importantly, TriCurin, formulated as a liposomal nanoformulation, has emerged as a leading HPV-specific phytochemical, demonstrating substantial preclinical efficacy in relevant cell lines and xenograft models. This advancement underscores the potential of combining nanoparticle technology with phytochemicals to improve treatment outcomes for patients with HPV+ OPSCC. 

### 6.2. Polymeric Nanoparticles (PLGA and Chitosan-Based)

Encapsulation of phytochemicals in poly(lactic-co-glycolic acid) (PLGA) nanoparticles addresses significant challenges, including poor water solubility, limited bioavailability, and rapid metabolic degradation. This biocompatible and FDA-approved delivery system enables sustained release, protects plant-derived compounds from enzymatic degradation, and enhances targeted therapeutic delivery. 

Chitosan-based nanoparticles (CSNPs) are an innovative solution for delivering phytochemicals in HPV-positive cancers [212] Their biocompatibility and biodegradability make them highly effective nanocarriers, safeguarding therapeutic plant compounds from degradation. Furthermore, CSNPs enhance cellular uptake and help to overcome drug resistance. These attributes make them a promising targeted treatment option for cervical, oral, and head-and-neck cancers, potentially improving therapeutic outcomes. The quercetin–chitosan system is particularly noteworthy because this combination increases quercetin bioavailability in HPV+ OPSCC. Quercetin has demonstrated anti-HPV oncogenic activity by targeting E6/E7 viral proteins, and its delivery via chitosan nanoparticles directly addresses the bioavailability limitation in HPV-driven cancers. Chitosan/sulfonyl-ether-β-cyclodextrin (SBE-β-CD)-conjugated delivery systems for quercetin demonstrated a remarkable improvement in bioavailability in HPV-related cancer cells (HeLa cells), with IC50 values indicating increased cytotoxicity (43.55 μM). 

### 6.3. Nanoemulsions and Microemulsions

Nanoemulsions and microemulsions are oil-in-water or water-in-oil colloidal dispersions with droplet sizes typically ranging from 20–200 nm and, respectively, 10–100 nm, designed to solubilize hydrophobic phytochemicals. They enhance mucosal absorption in the oropharyngeal region, improve chemical stability during processing and digestion, and can be produced in liquid, gel, paste, or solid forms for flexible administration routes.

Curcumin nanoemulsion (Cur-NE) has demonstrated significant efficacy in reducing the mRNA expression levels of HPV E6 and E7 oncogenes, while simultaneously increasing the levels of p53 and Rb proteins in TC-1 (HPV positive) cells at concentrations lower than those required for free curcumin (*p* < 0.05). Additionally, Cur-NE exhibited markedly greater tumor suppression and growth inhibition in subcutaneous TC-1 tumors than free curcumin (*p* < 0.01) [213]. These results provide compelling evidence of a nanoemulsion’s potential to effectively target HPV oncoproteins, demonstrating verified efficacy in vivo.

### 6.4. Solid Lipid Nanoparticles (SLNs)

Solid lipid nanoparticles (SLNs) are colloidal carriers composed of a solid lipid matrix, including glycerides, fatty acids, and waxes. These particles are typically 10 to 1000 nm in size and are stabilized by surfactants. In the context of oropharyngeal squamous cell carcinoma (OPSCC), SLNs offer a significant advantage over nanoemulsions and polymeric nanoparticles due to their combination of solid-state chemical protection and mucoadhesive compatibility. They protect labile phytochemicals (EGCG, curcumin, resveratrol) from enzymatic/oxidative degradation, prolong mucosal drug levels without repeated dosing, and are safe for long-term locoregional treatment. Incorporated in gels, sponges, or films, they can be delivered directly to the oropharynx and tonsils.

Research has demonstrated that curcumin-encapsulated lipidic nanoconstructs (CLEN-SLN) [214] achieve an impressive 69.78-fold increase in oral bioavailability relative to free curcumin, and a ninefold increase compared to a commercially available formulation (CurcuWIN®). Furthermore, this formulation enhances the solubility of curcumin by a factor of 1.4 million, compared to its intrinsic solubility of 11 ng/mL, exhibiting zero-order release kinetics and a 104-fold increase in stability at a pH of 6.8.

Curcumin-loaded SLNs reduced tumor growth more effectively than free curcumin, suppressed key pro-survival proteins (XIAP and Mcl-1) and inflammatory cytokines (IL-6 and TNF-α), and showed additive effects with chemotherapy. 

The local application of curcumin to the oral mucosa using a mucoadhesive sponge containing curcumin solid lipid nanoparticles has been identified as an effective strategy for treating precancerous oral lesions [215]. This treatment regimen involves a low dose of 6 mg per day for 6 weeks, resulting in decreased pain and accelerated healing of these lesions. These findings represent a noteworthy advancement in the clinical translation of phytochemical SLNs for oropharyngeal mucosal applications [216].

### 6.5. Cyclodextrin Inclusion Complexes

Cyclodextrins (CDs) form host–guest inclusion complexes with phytochemicals, encapsulating them within their hydrophobic cavity to improve aqueous solubility [217]. CD complexes enhance the loading capacity and carriage of plant bioactive constituents, protect against chemical degradation, and increase bioavailability for targeted delivery.

The strongest evidence related to HPV (human papillomavirus) comes from two specific formulations. The RES/HP-β-CD complex (RHSD) has been shown to significantly downregulate the HPV oncogenes E6 and E7 in vivo while upregulating the levels of P53 and Rb1. This approach demonstrated greater efficacy on these proteins compared to free resveratrol, achieving a 438.6-fold increase in solubility and transforming the crystalline structure of resveratrol into an amorphous form. Notably, this is the only cyclodextrin-phytochemical formulation with direct evidence of in vivo HPV oncoprotein suppression to date.

For curcumin-cyclodextrin complexes, the established in vivo model is the TC-1 system. When administered orally, these complexes reduced the size of implanted lung tumors in orthotopic settings. In vitro experiments showed impaired tumor cell proliferation and increased apoptosis, while the combination of curcumin-CD and gemcitabine enhanced therapeutic effects in C57Bl/6 mice. TC-1 cells express HPV16 E6/E7, making this study directly relevant to HPV+ OPSCC.

The SBE-β-CD/quercetin inclusion complex system demonstrated the best results in HMW chitosan formulations for HPV-related cervical cancer cells, showing a remarkable improvement in quercetin bioavailability. This approach is directly translatable to HPV- OPSCC, given the shared viral oncogenic mechanisms.

### 6.6. AI-Assisted Nanoparticle Design

Artificial Intelligence and Machine Learning are now being integrated into phytochemical profiling and nanoparticle design, offering predictive algorithms to improve green synthesis methods and optimization of loading efficiency.

Rapid assessment of plant extracts using high-throughput omics could accelerate the identification of optimal nanocarrier formulations for HPV+ OPSCC-specific phytochemical delivery. Artificial intelligence is reshaping the discovery of natural product-derived antitumor agents by enabling faster compound screening, more reliable pharmacological predictions, and more efficient optimization throughout the drug discovery process. By combining machine learning and deep learning approaches with multi-omics data and advanced bioinformatics tools, AI has greatly expanded our ability to explore the chemical diversity of phytochemicals and to identify candidates with genuine anticancer potential [218].

When integrated with metabolomic, proteomic, and cheminformatic analyses, AI-driven strategies provide a more systematic and informed approach to prioritizing bioactive compounds and understanding their possible mechanisms of action. This represents a meaningful shift away from traditional trial-and-error screening toward more targeted and data-driven phytochemical discovery.

Addressing these challenges will require improved data curation practices, the development of transparent and explainable AI models, and closer collaboration between computational scientists, experimental researchers, and clinicians.

Overall, AI-driven phytochemical screening stands out as both a necessary and promising strategy for developing new chemotherapeutic agents. As AI technologies mature and become more accessible, their thoughtful integration, supported by experimental validation and clinical insight, is likely to play an increasingly important role in advancing precision oncology and natural product–based cancer therapies. Challenges related to regulatory guidelines, scalability, and long-term safety must still be addressed to fully realize their clinical potential.

## 7. Clinical Translation Status

The main challenges to completing clinical trials include: (1) regulatory classification issues for natural products with multiple compounds; (2) variability in bioavailability that requires standardized formulations; (3) absence of validated HPV-specific molecular biomarkers for response monitoring; (4) funding challenges for compounds that cannot be patented.

Therefore, despite the depth of preclinical evidence, clinical trial data specifically for HPV-positive oral cancer remain limited. The following represent the most advanced translational positions.

### 7.1. Curcumin in HNSCC and HPV+ OPSCC

There are multiple Phase I/II trials with curcumin as an adjunct to chemo/radiotherapy: principal safety and tolerability findings are positive, while efficacy endpoints are mixed, largely limited by bioavailability (Safety and Efficacy of APG-157 in Head and Neck Cancer, NCT05312710, available online at https://clinicaltrials.gov/study/NCT05312710, accessed on 10 May 2026).

### 7.2. Polyphenon E (EGCG) for Oral Leukoplakia

A Phase I/II trial at MD Anderson shows acceptable safety and signs of efficacy in reducing premalignant lesions [219].

### 7.3. Veregen (Sinecatechins/EGCG)

Sinecatechins are FDA-approved for HPV-related external genital warts [220,221,222]. Recent clinical trials showed that Veregen^®^, a topical ointment containing 55–72 mg of EGCG, is a safe and effective treatment for vulval warts, benign lesions caused by low-risk HPV strains. A phase II randomized controlled trial (RCT) was conducted to assess whether Veregen^®^ is also effective against usual-type vulvar intraepithelial neoplasia (uVIN), a premalignant lesion associated with high-risk HPV infection. Patients eligible for the study were randomized to receive either Veregen^®^ or a placebo ointment (applied three times daily for 16 weeks), with follow-ups at 2, 4, 8, 16, 32, and 52 weeks. Twenty-six patients participated; all 13 treated with Veregen^®^ exhibited either a complete (n = 5) or partial (n = 8) clinical response (CR). The Veregen^®^ group showed a significant improvement in CR compared to the placebo group (*p* = 0.0026). No differences were observed in histological response or toxicity between the groups [223].

### 7.4. Silymarin for Radiotherapy-Induced Mucositis

Clinical observations (420 mg/day or 70 mg/5 mL oral nano-Silymarin solution) showed a significant reduction in the onset and severity of grade 3–4 mucositis [224,225,226].

Clinical evidence supports the mechanistic insights; silymarin at 420 mg per day decreased the severity and delayed the onset of radiotherapy-induced oral mucositis in patients with head and neck cancer—a highly relevant aspect, as mucositis is the most debilitating acute toxicity associated with radiotherapy for OPSCC [194,224,225,226,227,228].

Additionally, systemic administration of 140 mg three times daily showed a trend toward reducing chemotherapy-induced hepatotoxicity, further endorsing silybin’s role as a protective adjunct during standard treatments [193,229,230].

### 7.5. I3C for Recurrent Respiratory Papillomatosis

I3C is clinically used in adult patients as an adjunct to surgical resection; 200 mg/day for 3–4 years provides direct antiviral evidence against HPV in mucosal tissue [183].

Furthermore, the drug Epigalin^®^ (a combination of 400 mg I3C and 90 mg EGCG) is an effective oncoprotective, normalizing the balance of estrogen and contributing to the elimination of HPV [231].

The combination of 200 mg of indole-3-carbinol and 60 mg of trans-resveratrol for preventing persistent HPV infection and treating cervical intraepithelial neoplasia shows significant promise [185]. However, further research is needed to determine the most effective application methods.

Plant constituents for managing outcomes after surgery, chemotherapy, and radiotherapy are shown in Table 5.

Plant-based constituents can play a preventive role in HPV-ONSCC by directly inhibiting HPV-induced papillomatosis (Table 6).

## 8. Current Key Findings

Most plant bioactive compounds analyzed in the present review have demonstrated significant anti-HPV effects and anticancer activity against HPV-induced oropharyngeal and cervical cancers in preclinical studies.

### 8.1. Mechanistically Coherent Antiviral Targeting

Multiple compounds (curcumin, EGCG, berberine, tanshinone IIA, resveratrol) converge on the same HPV oncogenic axis—E6/E7 oncoprotein degradation and p53/Rb restoration—providing a biologically plausible and consistent mechanistic rationale.

### 8.2. Regulatory Proof of Concept

Sinecatechins (Veregen^®^) represents the first FDA-approved botanical drug for HPV-driven disease, demonstrating that plant-derived anti-HPV therapeutics can reach the highest regulatory standard.

### 8.3. Clinical RCT Evidence Supports Treatment Outcomes

Specifically for oral mucositis, multiple independent RCTs (curcumin, honey, silymarin, SAMITAL) and a 2024 systematic review/meta-analysis provide strong clinical evidence that several plant agents can reduce a key severe complication of HPV+ OPSCC treatment.

### 8.4. Immune Modulation in Immunocompetent Models

TriCurin studies using the TC-1 syngeneic model demonstrated that the anti-tumor effect depends on TAM repolarisation and CD8+ T cell recruitment—an immune mechanism absent in xenograft models, providing additional translational weight.

### 8.5. Favorable Safety Profiles

The overwhelming majority of plant compounds reviewed show no significant toxicity to normal cells at therapeutic doses, with several (silymarin, honey, aloe vera) demonstrating better tolerability than standard pharmacological agents in direct comparisons.

### 8.6. Synergistic Combination Evidence

Combinations (TriCurin, luteolin + asiatic acid, resveratrol + cisplatin) consistently outperform single agents in vitro and in vivo, suggesting combination phytotherapy or phyto-chemotherapy synergy may be more clinically relevant than monotherapy.

### 8.7. Coherent OPSCC Prevention Pathway

The mechanistic link between treating HPV papillomatosis at any site (genital, cervical, respiratory) and reducing oropharyngeal cancer risk is well-supported: shared HPV 16/18 strains, E6/E7-mediated carcinogenesis, and systemic viral load reduction all point toward a prevention cascade.

### 8.8. Nanoformulation Progress

Nanoparticle delivery systems (CUR-LCNPs, nano-silymarin, liposomal curcumin) are actively addressing the bioavailability limitations of polyphenols, with several showing superior efficacy to free compounds in head-to-head comparisons.

### 8.9. Growing Recency of Evidence

Several key studies were published in 2023–2025—including the only study using primary HPV+ OPSCC patient-derived cell lines [254], the most comprehensive tanshinone + curcumin W12 model study [196], and the most recent sinecatechins RCT [246], demonstrating an active and expanding research field.

**Figure 4:**
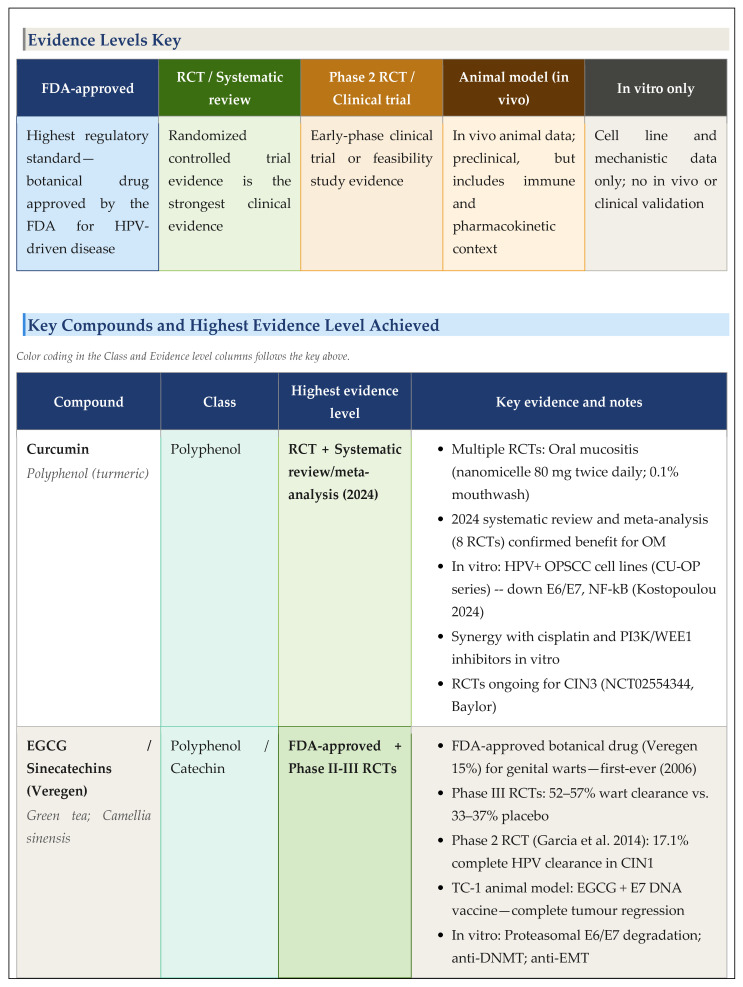

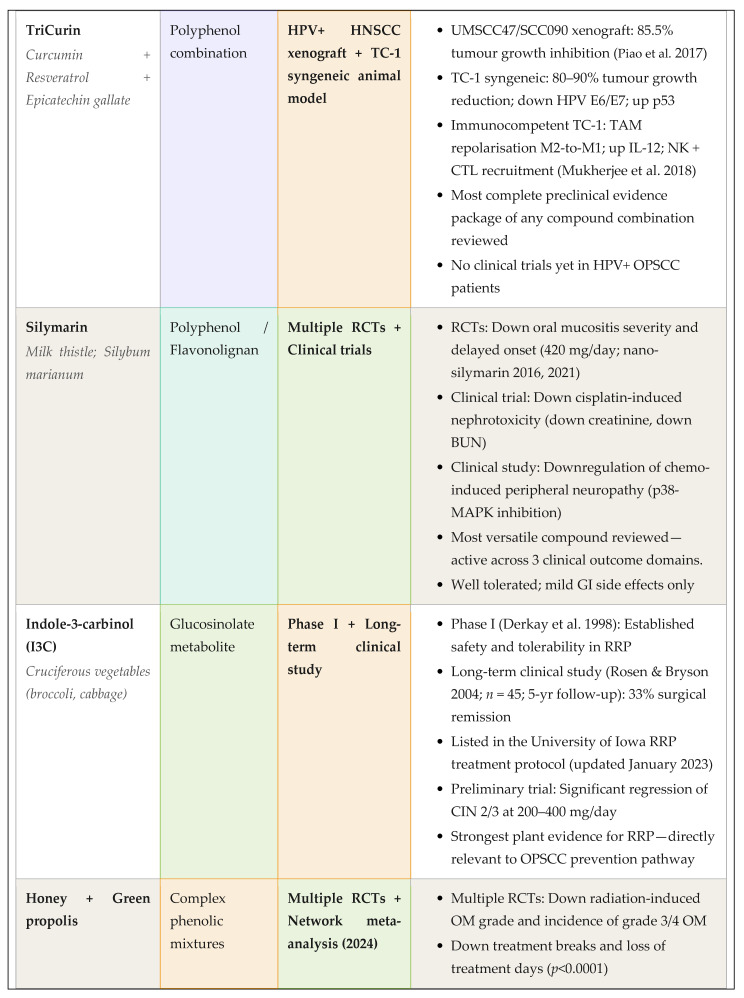

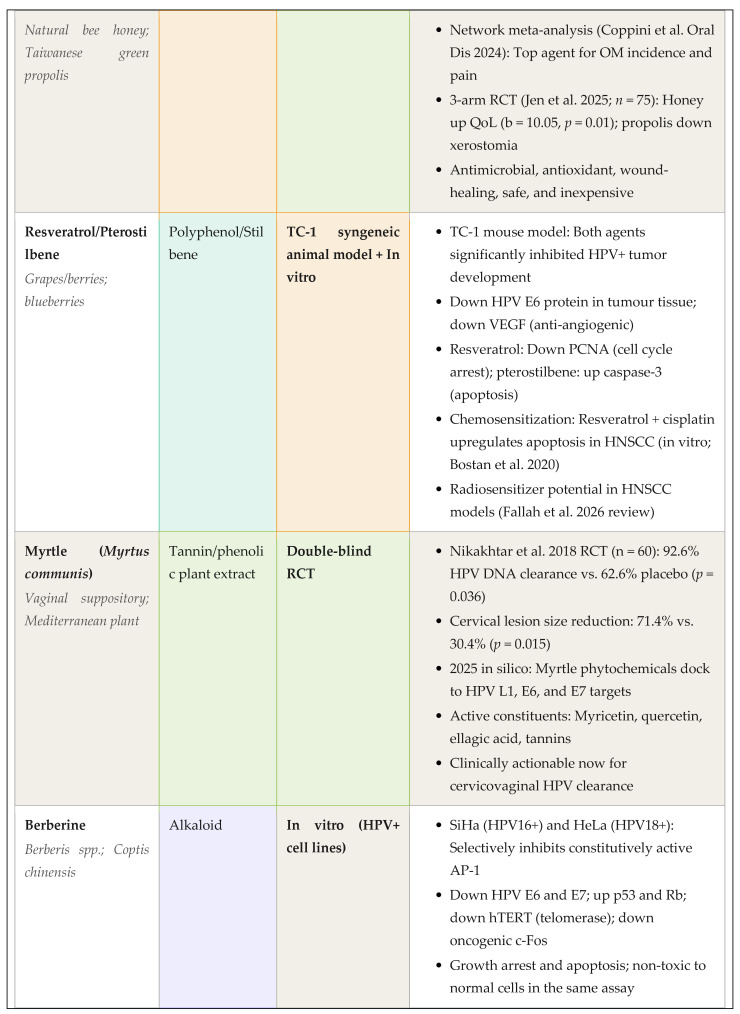

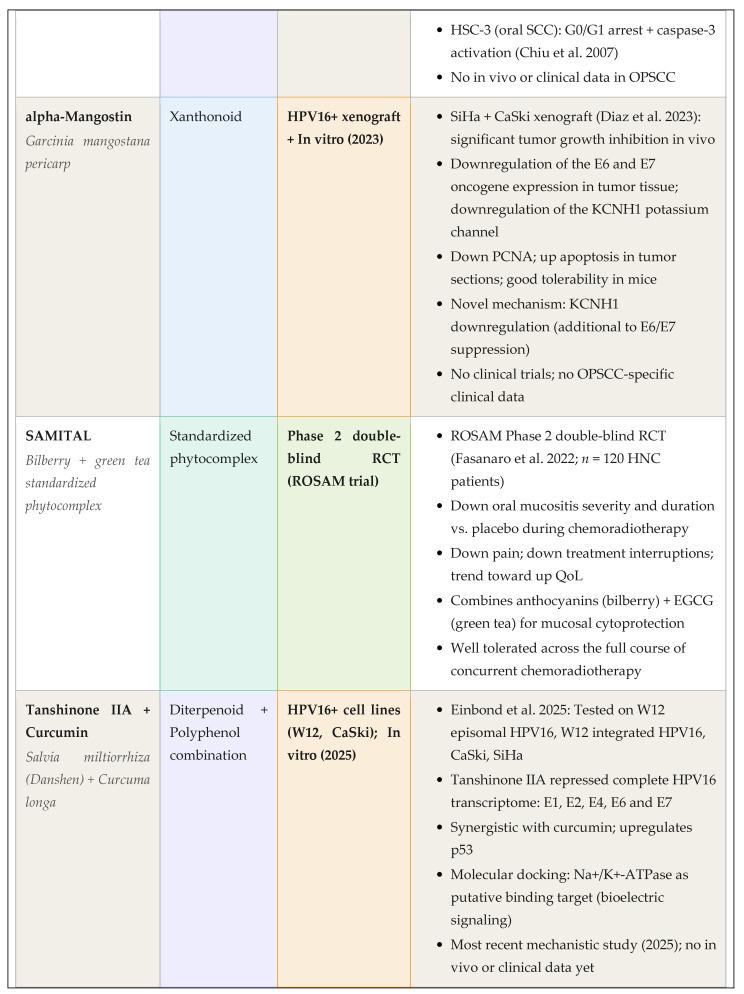

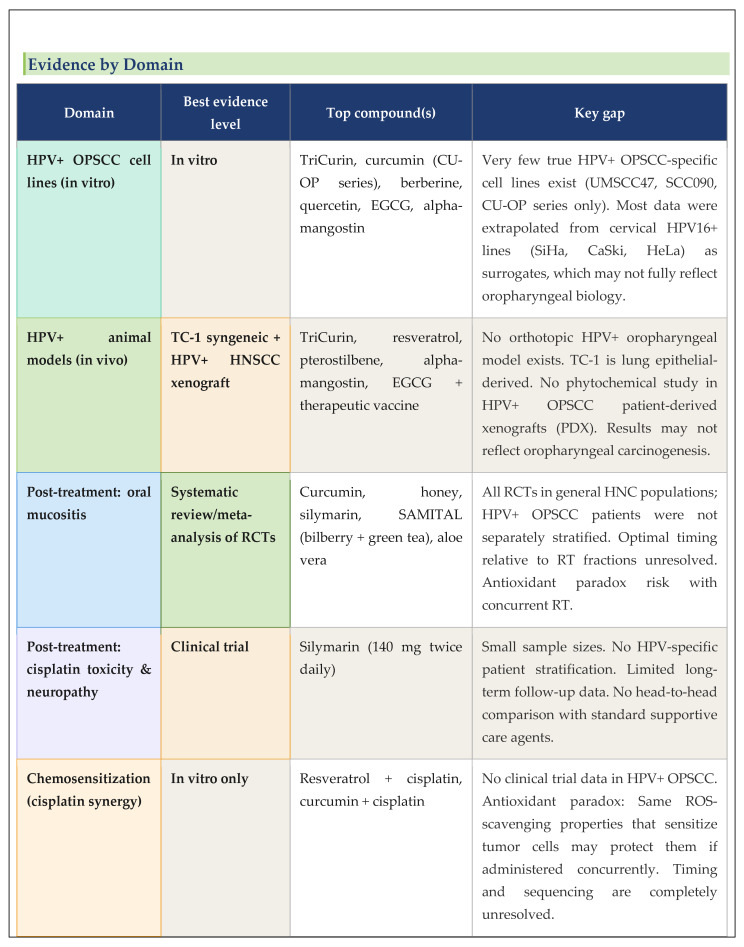

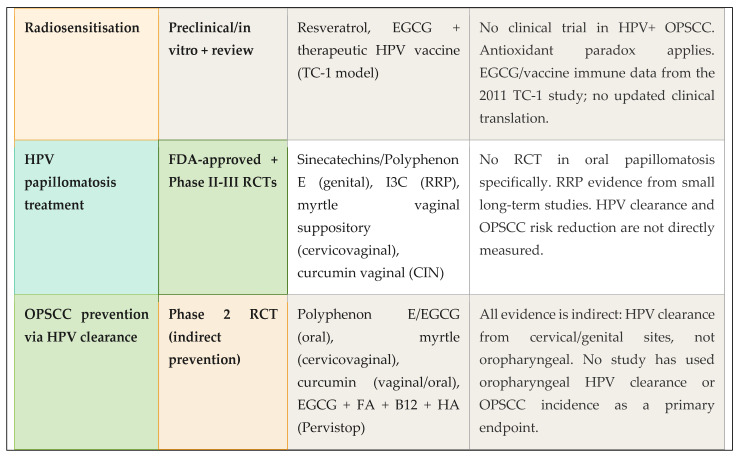
Evidence-based plant products with potential benefits in HPV+ OPSCC.

In summary, these phytochemicals may provide a scientifically supported, mechanistically consistent, and toxicologically safe adjuvant approach for HPV-positive oral cancer (Figure 4).

## 9. Limitations and Critical Gaps

### 9.1. No Dedicated HPV+ OPSCC Clinical Trial for Any Compound as an Anti-Cancer Therapy

Despite abundant in vitro and animal model data, no compound has completed a Phase II or III randomized controlled trial in HPV+ OPSCC patients as the target population, with tumor response as the primary endpoint. The entire anti-cancer evidence base remains preclinical.

### 9.2. Cell Line Model Gap

The majority of in vitro data has been generated using HPV16+ cervical cancer lines (SiHa, CaSki, HeLa) as surrogates for HPV+ OPSCC biology. Only a small number of true HPV+ OPSCC cell lines exist (UMSCC47, UPCI: SCC090, CU-OP series), and most have been used in only one or two published studies. Results from cervical lines may not fully translate to oropharyngeal biology.

### 9.3. Bioavailability Challenge

Polyphenols such as curcumin, quercetin, and resveratrol have notoriously low oral bioavailability (curcumin <1% in some estimates), rapid first-pass metabolism, and poor solubility. The in vitro concentrations required for HPV E6/E7 suppression are often far higher than physiologically achievable plasma levels. Nanoformulations are promising but remain largely in early-phase development.

### 9.4. Antioxidant Paradox During Treatment

The same ROS-scavenging properties that protect normal tissues from radiotherapy and cisplatin toxicity may, in theory, protect tumor cells from treatment-induced killing. The optimal timing of phytochemical administration relative to RT/CT remains unresolved, and concurrent use of antioxidant-rich supplements during active treatment carries theoretical risks, with limited supporting clinical safety data.

### 9.5. Animal Model Limitations

The TC-1 syngeneic model (the most widely used HPV+ preclinical model) was derived from mouse lung epithelial cells, not oropharyngeal tissue. No orthotopic HPV+ oropharyngeal model exists. No phytochemical study has been published using patient-derived xenografts (PDX) from HPV+ OPSCC. Results from TC-1 or SiHa/CaSki xenograft models may not reflect the biology of oropharyngeal carcinogenesis.

### 9.6. Post-Treatment Clinical Data Are Not HPV-Specific

All RCTs for mucositis (curcumin, honey, silymarin, SAMITAL, aloe vera) were conducted in general head and neck cancer populations. HPV+ OPSCC patients have a distinct biology, better prognosis, and different treatment responses from HPV-negative HNSCC. Subgroup analyses by HPV status are rarely reported.

### 9.7. Standardization and Reproducibility Remain Difficult

The phytochemical content of plant extracts can vary greatly depending on species, growing conditions, and preparation methods. Additionally, most studies lack detailed phytochemical profiling of the extracts used. As a result, comparing studies that use the same nominal compound, such as curcumin, is challenging.

### 9.8. Small Sample Sizes and Publication Bias

Many clinical trials in this space have low enrolment (n = 30–75), are conducted at single centers, and are predominantly from specific geographic regions. Positive results are more likely to be published, inflating apparent efficacy. Most combination studies remain in vitro with no in vivo validation.

### 9.9. Moderate Herb–Drug Interaction Risk

Compounds such as curcumin, resveratrol, EGCG, and berberine are known modulators of CYP450 and P-gp. In patients receiving cisplatin, 5-FU, or immunotherapy (pembrolizumab—standard of care in recurrent/metastatic HPV+OPSCC), pharmacokinetic herb–drug interactions could alter efficacy and toxicity in unpredictable ways. This risk is largely unstudied in the OPSCC context.

## 10. Essential Considerations and Future Directions

Plant bioactive constituents represent a biologically well-founded, mechanistically coherent, and pharmacologically active field in HPV+ OPSCC research. The evidence base spans a broad spectrum—from in vitro antiviral activity to clinical trials for treatment toxicity management to an FDA-approved compound for HPV-driven lesions. However, the translation gap between preclinical promise and clinical proof remains substantial, and no plant-derived agent can yet be recommended as an anti-cancer therapy specifically for HPV+ OPSCC based on current evidence.

The highest-quality, most clinically relevant evidence supports the use of plant agents to improve outcomes in severe conditions. Curcumin (multiple RCTs + meta-analysis), honey and propolis (RCTs + network meta-analysis 2024), silymarin (RCTs for mucositis, a clinical trial for nephrotoxicity and neuropathy), and SAMITAL (Phase 2 RCT) are a group of agents with enough clinical evidence to justify their inclusion in supportive care protocols—not as cancer treatments, but as adjuncts that lower treatment-related morbidity.

TriCurin (curcumin + resveratrol + epicatechin gallate) has the most comprehensive preclinical evidence package: HPV+ HNSCC cell line data, TC-1 syngeneic model, HPV+ HNSCC xenograft (85.5% inhibition), and a mechanistic immunological study demonstrating TAM reprogramming. EGCG and its combinations (Polyphenon E, Pervistop^®^) possess both mechanistic depth and clinical trial support across multiple sites and HPV disease states. Both are strong candidates for early-phase clinical trials in HPV+ OPSCC.

The prevention pathway—which involves clearing HPV 16/18 from genital, cervical, and respiratory sites to decrease oropharyngeal exposure and viral load—is the most immediately applicable clinical strategy. Sinecatechins (FDA-approved), indole-3-carbinol (with clinical evidence in RRP and included in the Iowa protocol), and myrtle/Polyphenon E (used in cervicovaginal RCTs) are compounds where clinical use can currently be justified, and where the mechanistic connection to lower OPSCC risk is most clear.

The following research gaps should be urgently addressed:

○ Dedicated HPV+ OPSCC clinical trials: Phase I/II trials of curcumin (alone or as TriCurin), EGCG, and berberine in HPV+ OPSCC patients are urgently needed. Trial design should stratify by HPV status and incorporate HPV E6/E7 suppression as a pharmacodynamic biomarker.
○ Orthotopic animal model development: A syngeneic, orthotopic HPV+ oropharyngeal tumor model in immunocompetent mice would transform the translational value of preclinical phytochemical studies. The current TC-1 model is an insufficient surrogate.
○ HPV-stratified subgroup analysis in treatment RCTs: Future clinical trials testing plant agents for mucositis or other treatment outcomes should report HPV status and conduct pre-planned subgroup analyses to determine whether effects differ by HPV positivity.
○ Bioavailability solutions at scale: Nano-formulation strategies (liposomal, polymeric NP, nano-micelles) for curcumin, EGCG, and resveratrol require Phase I pharmacokinetic trials in OPSCC patients to confirm that oropharyngeal tissue levels are therapeutically relevant.
○ Herb–drug interaction profiling: Systematic assessment of CYP450 and P-glycoprotein interactions for the leading compounds combined with pembrolizumab (now standard of care in recurrent HPV+ HNSCC) is an unmet safety research need.

Future research on the design of rational combination regimens should be validated using both computational methods and HPV-positive HNSCC-specific in vivo models, with orthotopic tumor models preferred over subcutaneous xenografts for oropharyngeal disease. Additionally, prospective biomarker-enriched clinical trials in HPV-positive OPSCCs—using HPV E6/E7 suppression, p16 restoration, and PD-L1 modulation as pharmacodynamic endpoints—could be vital for further investigation. Future studies might analyze combinations with pembrolizumab or nivolumab, given that EGCG, berberine, and curcumin all reduce PD-L1 expression and could serve as chemosensitizers for checkpoint immunotherapy. Moreover, advanced research could examine epigenetic interactions between phytochemicals and HPV integration sites, especially regarding HDAC inhibitors (I3C), which may derepress integration-silenced viral genomes.

## Figures and Tables

**Table 1:** The main differences between HPV-positive and HPV-negative HNSCC [8,9,10,11,12,13,14,15,16,17].

Feature	HPV-Positive HNSCC	HPV-Negative HNSCC
Primary risk factors	HPV (esp. HPV-16), sexual behavior	Tobacco, alcohol
Specific patient	Younger, male, non-smoker	Older, smoker/drinker
Anatomical site	Tonsil, base of tongue (oropharynx)	Oral cavity, larynx
Key oncoproteins	E6, E7 (viral)	Mutant TP53, EGFR overexpression
p16 status	Overexpressed (high)	Low/absent
Histology	Non-keratinizing, basaloid	Keratinizing SCC
AJCC staging	Separate, favorable system (8th ed.)	Conventional staging
TME	Immunogenic, T-cell rich	Immunosuppressive
Radiosensitivity	High	Lower
De-escalation therapy	Active area of investigation	Not applicable
Prognosis	Significantly better	Worse
Recurrence frequency	Lower	Higher
Prevention	Vaccination (HPV vaccine)	Tobacco/alcohol cessation

**Table 2:** Molecular Mechanisms of Plant Constituents in HPV+ OPSCC Cell Lines.

Constituent	Class	Cell Line(s)	Key Findings	Molecular Targets/Mechanisms	References
TriCurin (Curcumin + Resveratrol + Epicatechin gallate)	Polyphenol combination	UMSCC47; UPCI:SCC090 (HPV16+)	↓ Cell viability, clonogenic survival, tumorsphere formation, significant apoptosis, inhibited tumor growth by 85.5% in vivo	↓ HPV16 E6/E7 oncoproteins; ↑ p53; caspase activation	[151]
		HPV+ TC-1; UMSCC47 (HPV16+)	↓ HPV E6; eliminates HPV+ cancer cells; ↑ p53 accumulation; intralesional injection → 80–90% ↓ tumor growth in mice	↓ HPV E6 oncoprotein; ↑ p53 and acetyl-p53; caspase-3 activation	[149]
		HPV+ TC-1 (HPV16+)	Repolarises M2 → M1 tumour-associated macrophages; triggers IL-12 signalling; recruits NK cells and cytotoxic T lymphocytes; immune-mediated tumor suppression	TAM repolarisation; ↑ IL-12; NK/CTL recruitment; PD-L1 pathway modulation	[145]
Curcumin	Polyphenol	CU-OP-2; CU-OP-3; CU-OP-20 (HPV+ OPSCC primary lines)	Dose-dependent ↓ viability and proliferation; synergistic effects with PI3K inhibitor BYL719 and WEE1 inhibitor MK-1775	CDC27 targeting; PI3K/AKT/mTOR inhibition; CDK4/6 pathway; WEE1 G2/M checkpoint	[169]
		93VU147T (HPV16+ oropharyngeal)	Suppresses AP-1 and NF-κB transcription factor activity; selectively reduces HPV16 E6 oncogene transcription	↓ AP-1 and NF-κB activity; ↓ HPV16 E6 mRNA; anti-inflammatory signalling	[170]
Curcumin + Metformin	Polyphenol	UMSCC47; UMSCC1 (HPV+ and HPV−)	Both agents slow proliferation and induce apoptosis; curcumin effects are more pronounced in HPV lines; metformin is more effective in HPV+ lines; no synergy observed	↓ Proliferation; ↑ apoptosis; AMPK pathway (metformin); multiple HNSCC biomarkers affected	[170,171,172]
*Lycium barbarum* phenolic extract (flavonols, flavan-3-ols, hydroxycinnamic acid amides)	Phenolic plant extract	UPCI:SCC090 (HPV16+); CAL27	G_0_/G_1_ arrest and S-phase accumulation in SCC090; ↓ E6/E7 mRNA (*p* < 0.05); ↑ p53 protein; ↓ Ki-67 and Bcl-2 expression	Cell cycle arrest; ↓ HPV E6/E7 oncogene expression; p53 stabilisation; anti-proliferative and immunomodulatory	[173]
Berberine	Alkaloid	HSC-3 (oral/oropharyngeal SCC)	Dose- and time-dependent irreversible growth inhibition; G_0_/G_1_ cell cycle arrest; apoptosis via caspase-3 activation and ↓ mitochondrial membrane potential	Caspase-3 activation; G_0_/G_1_ arrest; mitochondrial pathway apoptosis; ↓ MMP	[174]
Quercetin	Polyphenol/Flavonol	UMSCC-47 (HPV+, tongue)	↓ Cell viability in vitro; Ca^2+^ response via T2R14 bitter taste receptor; mitochondrial depolarisation; cytotoxicity in ex vivo tumor slices	T2R14 agonism; intracellular Ca^2+^ signalling; mitochondrial membrane depolarisation; apoptosis induction	[157]
Fisetin (flavonoid)	Polyphenol/Flavonol	HPV-18 E6/E7 models (in silico + OSCC lines)	Lead photosensitizer targeting HPV-18 E6 and E7 oncoproteins via molecular docking; established anti-proliferative activity in OSCC cell lines.	HPV-18 E6/E7 oncoprotein binding; photodynamic inhibition; anti-proliferative, antiviral, antioxidant	[175]
EGCG (epigallocatechin-3-gallate; green tea)	Polyphenol/Catechin	SCC-9 (OSCC); FaDu (head & neck SCC)	Near-complete inhibition of invasion; ↓ MMP-2 and uPA; anti-EMT (↓ Snail-1, vimentin); ↓ FAK/Src phosphorylation; inhibited DNMT activity up to 80%	MMP-2/9 inhibition; anti-EMT; DNMT inhibition (epigenetic); FAK/Src pathway; reactivates silenced tumour suppressors	[176]
Proanthocyanidins (PAC)	Polyphenol/Tannin	HPV-transfected OSCC; SiHa (HPV+)	PAC inhibits the proliferation of HPV-transfected OSCC cells dose-dependently; tested in cervical carcinoma and non-cancerous lines	↓ Cell proliferation; anti-HPV; modulates p53 and Rb pathway activity	[177]

↓—inhibition; ↑—activation.

**Table 3:** Molecular Mechanisms of Plant Constituents in HPV-Cervical Cancer Cell Lines.

Constituent	Class	Cell Line(s) [HPV Status]	Key Findings	Molecular Targets	Reference
Tanshinone IIA + Curcumin	Combination (diterpenoid + polyphenol)	W12 episomal HPV16; W12 integrated HPV16; CaSki; SiHa	Both agents inhibit W12 and cervical cancer cell growth; TanIIA represses HPV16 E1, E2, E4, E6, E7 transcripts; ↑ p53; curcumin + TanIIA synergistic; molecular docking indicates Na+/K+-ATPase binding	↓ HPV16 E1/E2/E4/E6/E7; ↑ p53; Na+/K+-ATPase bioelectric signalling	[196]
Curcumin	Polyphenol	HeLa (HPV18+); CaSki (HPV16+)	Dose- and time-dependent ↓ cell viability; ↑ apoptosis (flow cytometry); ↓ mitochondrial membrane potential (JC-1); ↓ invasion and migration (transwell + wound-healing); selectively ↓ E6 > E7; ↓ Bcl-2/N-cadherin/vimentin; ↑ Bax/cleaved caspase-3/E-cadherin	↓ HPV E6 (primarily) and E7; ↑ p53; mitochondrial apoptosis; anti-EMT	[197]
α-Mangostin (*Garcinia mangostana*)	Xanthonoid	SiHa (HPV16+); CaSki (HPV16+); HeLa (HPV18+); C33a (HPV−)	Concentration-dependent ↓ cell proliferation in all HPV+ lines; G1/S arrest; ↓ E6 and E7 oncogene expression; ↓ KCNH1 potassium channel gene; significant tumor growth inhibition in the mouse xenograft model	↓ HPV E6/E7 oncoproteins; ↓ KCNH1; G1/S arrest; antiviral + antineoplastic	[187]
Luteolin + Asiatic acid (flavonoid + triterpenoid)	Combination	CaSki (HPV16+); HeLa (HPV18+)	Combination is more effective than either agent alone; sub-G1 phase arrest; caspase-mediated intrinsic apoptosis; ↑ mitoROS; ↓ invasion and migration (wound-healing assay); inhibited xenograft tumor growth in vivo with minimal toxicity to normal cells	↓PI3K/AKT/p70S6K; ↓ JNK/p38 MAPK; ↓ FAK/integrin β1/paxillin; ↑ ERK; caspase cascade activation	[198]
EGCG (green tea; *Camellia sinensis*)	Polyphenol/Catechin	SiHa (HPV16+); CaSki (HPV16+); HeLa (HPV16/18+)	EGCG nanoformulations ↓ cell viability dose- and time-dependently; G2/M arrest in SiHa and HeLa; modulates oncomiRs (miR-210↑, miR-29a, miR-203, miR-125b); inhibits TGF-β-induced EMT; regulates DNA methylation via DNMT inhibition	↓ DNMTs (epigenetic); G2/M checkpoint arrest; anti-EMT; miRNA modulation; TGF-β pathway	[199]
		HFK-HPV18 (keratinocytes); VIN (HPV18+)	↓ Proliferation in monolayer and organotypic raft culture stimulates proteasomal degradation of E6 and E7 oncoproteins; ↑ p53 and Rb; suppresses E4 late viral protein; blocks productive HPV replication in differentiating keratinocytes	Proteasomal E6/E7 degradation; ↑ p53/Rb; ↓ HPV late protein E4; blocks productive viral replication	[200]
Quercetin (flavonol; onions, capers)	Polyphenol/Flavonol	SiHa (HPV16+); HeLa (HPV18+)	Selective toxicity toward HPV+ lines vs. normal fibroblasts (7–8× higher IC50 in normal cells); G2 phase arrest; ↑ Bax/Bcl-2 ratio; caspase 3/7 activation; ↑ p53 in an E6 expression-independent manner; apoptotic morphological changes	G2 arrest; ↑ p53 (E6-independent); ↑ Bax/↓ Bcl-2; caspase 3/7 activation; selective HPV+ cytotoxicity	[201]
EGCG (green tea; *Camellia sinensis*)	Polyphenol/Catechin	SiHa (HPV16+); CaSki (HPV16+); HeLa (HPV16/18+); C33A (HPV−)	Dose- and time-dependent ↓ proliferation; IC50 90.74 µg/mL (24h) in HeLa; modulates tumour-suppressive miRNAs: ↑ miR-210 and miR-29a in CaSki/SiHa; ↑ miR-125b and miR-203 in SiHa; distinct miRNA profiles by HPV subtype	miRNA modulation (miR-210, miR-29a, miR-203, miR-125b); ↓ proliferation; HPV subtype-specific epigenetic effects	[136]
Berberine (*Berberis* spp.)	Alkaloid	SiHa (HPV16+); HeLa (HPV18+)	Selectively inhibits constitutively active AP-1; dose- and time-dependent ↓ E6 and E7 oncogene expression; ↓ oncogenic c-Fos; ↑ p53 and Rb; ↓ hTERT (telomerase); growth arrest and apoptosis; no toxicity to normal cells	AP-1 inhibition; ↓ HPV E6/E7; ↑ p53/Rb; ↓ hTERT; c-Fos downregulation	[202]
Tanshinone IIA (*Salvia miltiorrhiza*; Danshen)	Terpenoid/Diterpenoid	CaSki (HPV16+); SiHa (HPV16+); HeLa (HPV18+); C33a (HPV−)	↓ Proliferation in all HPV+ lines; S-phase arrest; ↓ HPV E6 and E7 (mechanistic study in CaSki); modulates E6AP and E2F1; ↑ p53 accumulation and p53-dependent targets; ↓ Bcl-2/↑ Bax; caspase-3 activation; PARP cleavage; p53-mediated apoptosis	↓ HPV E6/E7; ↑ p53; S-phase arrest; E6AP/E2F1 modulation; Bcl-2/Bax; caspase-3; PARP cleavage	[203]
α-Mangostin (*Garcinia mangostana*)	Xanthonoid	SiHa (HPV16+); HeLa (HPV18+)	↓ Cell viability dose-dependently; loss of mitochondrial membrane potential; ↑ cytochrome C release; ↑ Bax/↓ Bcl-2; caspase-9/caspase-3 cascade; ↑ ROS → activates p38 MAPK via ASK1; tumour growth inhibition in xenograft model	ROS generation; ASK1/p38 MAPK activation; mitochondrial apoptosis; Bax/Bcl-2; caspase-9/3	[204]
Apigenin (parsley, chamomile)	Polyphenol/Flavone	SiHa (HPV16+); CaSki (HPV16+); HeLa (HPV18+); HaCaT (normal, control)	Selective cytotoxicity in all HPV+ lines vs. non-tumorigenic HaCaT; Annexin V+ apoptosis; ↑ ROS and H_2_O_2_; ↓ mitochondrial membrane potential; ↑ lipid peroxidation; ↓ migration and invasion	Oxidative stress (↑ ROS/H_2_O_2_); mitochondrial redox impairment; apoptosis; anti-migratory	[178]

↓—inhibition; ↑—activation.

**Table 4:** Molecular Mechanisms of Plant Constituents in HPV+ Cancer Animal Models.

Constituent	Class	Animal Model	Key In Vivo Findings	Mechanisms Confirmed In Vivo	Reference
TriCurin *(Curcumin + Resveratrol + Epicatechin gallate)*	Polyphenol combination	Model type: Immunocompetent mouse (TC-1) Detail: C57BL/6 mice implanted with TC-1 cells (HPV16 E6/E7/c-Ha-Ras+); intratumoral TriCurin injection	Intratumoral TriCurin repolarised M2 → M1 tumor-associated macrophages; ↑ IL-12 in tumor; recruited NK cells and CD8+ CTLs; immune-mediated tumor suppression confirmed in immunocompetent host only (not in nude mice), proving the immune mechanism is essential.	TAM repolarisation (ARG1 ↓, IL-10 ↓, iNOS ↑, IL-12 ↑); NK cell + CTL intratumoral recruitment; adaptive immune activation	[149]
		Model type: TC-1 syngeneic + HPV+ HNSCC xenograft Detail: (i) TC-1 HPV16+ tumors in C57BL/6 mice; (ii) UMSCC47 and UPCI: SCC090 xenograft in nude mice. Intratumoral/intralesional injection.	TC-1 tumors: 80–90% growth reduction vs. vehicle; UMSCC47/SCC090 xenografts: 85.5% tumour growth inhibition (*p* < 0.05, *n* = 7); ↓ E6/E7 in tumour tissue; ↑ p53, ↑ acetyl-p53 (activated), ↑ caspase-3; 4.7-fold greater E6 inhibition vs. curcumin alone; no adverse effects in tumour-naïve mice.	↓ HPV16 E6/E7 in vivo; ↑ p53 + acetyl-p53; caspase-3 activation; antitumour effect stronger than single agents	[151]
Resveratrol *(stilbene; grapes, berries)*	Polyphenol/Stilbene	Model type: TC-1 syngeneic Detail: C57BL/6 mice s.c. implanted with TC-1 cells (HPV16 E6/E7/c-Ha-Ras+); i.p. resveratrol treatment	Significantly inhibited TC-1 tumor development; ↓ E6 protein in tumor tissue; ↓ VEGF levels; tumor size reduction associated with cell cycle arrest mechanism (↓ PCNA); distinct mechanism from pterostilbene (growth arrest rather than apoptosis).	↓ HPV E6 oncoprotein in vivo; ↓ VEGF (anti-angiogenic); ↓ PCNA (cell cycle arrest); tumour growth suppression	[146]
Pterostilbene *(stilbene; blueberries; resveratrol analogue)*	Polyphenol/Stilbene	Model type: TC-1 syngeneic Detail: C57BL/6 mice s.c. implanted with TC-1 cells (HPV16 E6/E7/c-Ha-Ras+); i.p. pterostilbene; head-to-head with resveratrol in same study	Significantly inhibited TC-1 tumor development; ↓ E6 protein in tumor tissue; ↓ VEGF levels; tumor size reduction via apoptosis (↑ cleaved caspase-3); more potent than resveratrol in the same model; distinct mechanism (apoptotic rather than cell cycle arrest).	↓ HPV E6 oncoprotein in vivo; ↑ activated caspase-3 (apoptosis); ↓ VEGF; superior efficacy vs. resveratrol in same model	[205]
Curcumin-loaded liposomes *(PDT photosensitizer; liposomal nanoformulation)*	Polyphenol/Nanoformulation	Model type: VX2 rabbit (CRPV+ HNSCC model) Detail: CRPV-positive VX2 cells from New Zealand White rabbit (established animal model for papillomavirus-associated HNSCC); tested alongside UD-SCC-2 (HPV16+ HNSCC) and HeLa (HPV18+)	Curcumin liposomes + PDT: dose-dependent ↓ cell viability in VX2 (CRPV+), UD-SCC-2 (HPV16+), and HeLa; ↑ apoptosis; ↓ proliferation, migration, and colony formation in HPV-associated lines; liposomal formulation more effective than free curcumin; VX2 rabbit confirmed PDT sensitivity of papillomavirus-associated HNSCC.	PDT-mediated ROS generation and apoptosis; ↓ cell migration and colony formation; papillomavirus-specific sensitivity demonstrated; nanoformulation enhances delivery	[206]
Curcumin CUR-LCNPs *(lipid-coated polymeric nanoparticles; PDT)*	Polyphenol/Nanoformulation	Model type: HPV+ HNSCC panel (in vitro/translational) Details: 3 HPV+ HNSCC lines (incl. UD-SCC-2, HPV16+) and 3 HPV− lines; no pure in vivo xenograft endpoint but included for translational relevance	CUR-LCNPs (153 nm, 92.7% encapsulation) + PDT: significant tumor cell killing across all 6 HNSCC lines; no significant difference between HPV+ and HPV− groups (IC50 9.34 vs. 6.88 µM); superior to free curcumin; confirms suitability of curcumin PDT for HPV+ HNSCC regardless of HPV status.	ROS-mediated PDT cytotoxicity; mitochondrial depolarisation; nanoparticle uptake independent of HPV status; photosensitizer efficacy confirmed	[207]
α-Mangostin *(Garcinia mangostana)*	Xanthonoid	Model type: HPV16+ xenograft Detail: Athymic nude mice s.c. implanted with SiHa (HPV16+) or CaSki (HPV16+) cervical cancer cells	Significant inhibition of SiHa and CaSki xenograft tumor growth; ↓ E6 and E7 oncogene expression in tumor tissue; ↓ KCNH1 potassium channel gene; ↓ PCNA (proliferation marker); ↑ apoptosis in tumor sections; good tolerability in mice.	↓ HPV E6/E7 oncoproteins in vivo; ↓ KCNH1; ↓ PCNA; ↑ apoptosis; antineoplastic and antiviral activity confirmed in vivo	[204]
Luteolin + Asiatic acid *(flavonoid + triterpenoid combination)*	Combination (flavonoid + terpenoid)	Model type: HPV16+ xenograft Detail: Nude mice s.c. implanted with CaSki cells (HPV16+); oral/i.p combination treatment	Combination markedly inhibited CaSki xenograft tumor growth vs. single agents or control; minimal cytotoxicity to normal tissues; ↓ tumor weight and volume; PI3K/AKT and JNK/p38 pathway suppression confirmed in excised tumor tissue; superior to either agent alone.	↓PI3K/AKT/p70 S6K; ↓ JNK/p38 MAPK; ↓ FAK signalling; ↑ ERK; caspase cascade confirmed in vivo; low host toxicity	[198]
EGCG + E7-LAMP DNA vaccine *(EGCG as immunoadjuvant; green tea)*	Polyphenol/immunoadjuvant	Model type: TC-1 syngeneic (immunocompetent) Detail: C57BL/6 mice with established TC-1 tumours (HPV16 E7+); oral EGCG + Sig/E7/LAMP-1 DNA vaccination	Oral EGCG alone: modest antitumor effect. Combined with E7/LAMP DNA vaccine: complete tumor regression; ↑ E7-specific CD8+ cytotoxic T cells vs. vaccine alone; enhanced primary and memory T cell responses; no severe systemic toxicity (no hair loss, weight loss, or lymphopenia).	↑ CD8+ T cell-mediated antitumor immunity; immunoadjuvant synergy with therapeutic HPV vaccine; ↑ tumor-specific cytotoxicity in vivo	[208]
Apigenin + E7-HSP70 DNA vaccine *(flavone; parsley; chemosensitiser + immunoadjuvant)*	Polyphenol/Flavone	Model type: TC-1 syngeneic (immunocompetent) Detail: C57BL/6 mice with TC-1 tumours (HPV16 E7+); apigenin + E7-HSP70 DNA vaccine combination	Apigenin rendered TC-1 tumor cells more susceptible to E7-specific CTL lysis; combined with the E7-HSP70 DNA vaccine → the highest frequency of primary and memory E7-specific CD8+ T cells among all groups; potent therapeutic antitumor effects; apigenin enhanced apoptotic tumor cell death in a dose-dependent manner.	↑ MHC-I antigen presentation; ↑ E7-specific CD8+ T cell frequency; apoptosis induction; ↑ memory T cell response; potentiates therapeutic HPV immunization	[209]

↓—inhibition; ↑—activation.

**Table 5:** Plant constituents and their various mechanistic pathways in HPV+ OPSCC outcomes and therapy.

Constituent	Outcome Addressed	Evidence Level	Key Findings	Mechanistic Pathway
Curcumin *Nanomicelle capsules—SinaCurcumin^®^ 80 mg twice daily × 7 weeks* [232]	Oral mucositis	RCT	OM severity was significantly lower in the curcumin group vs. the placebo at weeks 1, 4, and 7 (*p* < 0.001); ↓ pain scores; effective for chemoradiotherapy-induced OM in HNC patients (n = 50 RCT)	↓ NF-κB and pro-inflammatory cytokines (TNF-α, IL-6); antioxidant mucosal protection; ↓ ROS-mediated tissue damage
Curcumin *Mouthwash 0.1% w/v and nanomicelle soft gel SinaCurcumin^®^ 40 mg—3× daily during RT* [233]	Oral mucositis	RCT	Both formulations ↓ radiation-induced OM severity and pain vs. placebo (n = 37 RCT, HNC); mouthwash showed earlier onset of effect; nanomicelle better at week 3; well tolerated during RT alone or chemoRT	↓ Oxidative stress at mucosal level; anti-inflammatory (NF-κB); wound-healing promotion; ↓ ulceration depth
Curcumin *Systematic review and meta-analysis of 8 RCTs + network pharmacology* [234]	Oral mucositis	SR/meta-analysis	Meta-analysis confirmed curcumin ↓ OM severity and pain in HNC patients receiving RT and/or CT; network pharmacology + molecular docking identified NF-κB, TP53, and AKT1 as key targets; evidence supports curcumin as an adjunct to standard OM care	NF-κB, TP53, AKT1, VEGF pathway modulation; anti-inflammatory + antioxidant dual mechanism confirmed computationally and clinically
Natural bee honey *Topical application during RT—20 mL swish and swallow before and after RT.* [235]	Oral mucositis	RCT	Multiple RCTs: ↓ Radiation-induced OM severity and incidence of intolerable grade 3/4 mucositis; ↓ treatment breaks and loss of treatment days (*p* < 0.0001); does not interfere with tumor RT response; 2024 network meta-analysis ranked honey among top agents for OM incidence and pain reduction	Antimicrobial (↓ secondary Candida/bacterial infection); antioxidant (flavonoids, phenolic acids); wound healing (↑ epithelial regeneration); anti-inflammatory (↓ prostaglandins)
Taiwanese green propolis *Flavonoid-rich bee product—oral rinse/swallow during and post-RT* [236]	Oral mucositis	RCT	3-arm RCT (n = 75 HNC patients; Jen et al. 2025): Propolis group ↓ patient-reported OM symptoms and ↓ xerostomia vs. usual care; honey group ↑ QoL (β = 10.05, *p* = 0.01) and ↓ OM severity; both superior to standard care across 12-week post-RT follow-up	Chrysin and caffeic acid phenethyl ester (CAPE): Anti-inflammatory (↓ IL-1β, TNF-α), antioxidant, antimicrobial; mucosal barrier protection and epithelial regeneration
Silymarin (milk thistle) *Silybum marianum—oral 420 mg/day (three divided doses); nano-silymarin 70 mg/5 mL* [224,228]	Oral mucositis	RCT	Oral silymarin 420 mg/day: Significantly ↓ reduced mucositis grade and delayed onset vs. placebo (*p* < 0.01); also ↓ oral pain and xerostomia scores; nano-silymarin RCT (2021) confirmed findings with improved bioavailability; safe, well-tolerated.	Anti-inflammatory (↓ NF-κB, TNF-α); antioxidant (↑ GSH, SOD); mucosal cytoprotection; ↓ radiation-induced ROS damage to epithelium
SAMITAL *Standardized bilberry (Vaccinium myrtillus) + green tea* *phytocomplex—oral rinse during chemoRT* [237,238]	Oral mucositis	RCT	Phase 2 double-blind RCT—ROSAM trial (n = 120 HNC patients): SAMITAL oral rinse ↓ OM severity and duration vs. placebo; ↓ pain; ↓ treatment interruptions; trend toward ↑ QoL; well tolerated across the full course of concurrent chemoradiotherapy	Anthocyanins (bilberry): mucosal integrity, anti-inflammatory; EGCG (green tea): antioxidant, antimicrobial; combined cytoprotective effect on oral epithelium
Aloe vera *Aloe barbadensis—gel; topical application 3× daily during and after RT* [239]	Oral mucositis	Clinical trial	Multiple clinical trials and a 2024 systematic review confirm aloe vera gel ↓ grade and progression of RT-induced OM in HNC; it has also been studied for xerostomia and burning mouth. Active RCT (NCT06381635, 2024) comparing aloe vera + Manuka honey vs. saline; salivary TGF-β1 and EGF assessed	Acemannan polysaccharides: ↑ Fibroblast growth + epithelial repair; ↑ salivary EGF; ↓ IL-1β; antimicrobial; ↓ mucosal oxidative stress
Silymarin/silybin B *Milk thistle—oral 140 mg twice daily; prophylactic use during cisplatin-based CT* [240]	Cisplatin toxicity	Clinical trial	Clinical trial: Silymarin prophylaxis ↓ of cisplatin-induced nephrotoxicity (↓ serum creatinine, ↓ BUN); safe and well-tolerated adjunct. Silybin B (active isoform) confirmed to ↓ apoptosis, DNA damage, and cell cycle arrest in cisplatin-treated neuronal cells (mouse model, 2022). Hepatoprotective effects have also been documented.	↑ GSH and SOD (renal antioxidant defence); ↓ cisplatin accumulation in renal tubular cells; ↓ oxidative DNA damage; hepatoprotective (↓ AST/ALT); neuroprotective (↓ p38-MAPK apoptosis)
Silymarin *Milk thistle—oral 140 mg twice daily; adjunct during/post chemotherapy* [193]	Peripheral neuropathy	Clinical trial	Clinical study: Silymarin 140 mg twice daily significantly improved chemo-induced peripheral neuropathy; ↓ pain scores; minimal side effects (mild GI symptoms only); protective against oxaliplatin- and cisplatin-induced neurotoxicity; p38-MAPK inhibition confirmed as protective mechanism	↓ p38-MAPK-mediated neuronal apoptosis; ↑ BDNF (brain-derived neurotrophic factor); ↓ oxidative stress in neural tissue; anti-neuroinflammatory (↓ microglial activation)
Resveratrol *Stilbene (grapes, berries)—preclinical and* in vitro *studies; HNSCC models* [241]	Radiosensitisation	Preclinical/in vitro	Resveratrol ↑ sensitivity of HNSCC cells to RT; ↓ NF-κB and JAK/STAT survival signaling post-irradiation; ↓ DNA repair capacity in tumor cells; pre/post-RT administration ↓ hematopoietic ROS, ↓ NOX4/COX-2; also protects normal tissue (antioxidant dual role); 2026 systematic review confirms radiosensitizing potential in HNSCC	↓ NF-κB, ↓ JAK/STAT; ↓ DNA repair (Ku70/Ku80) in tumour cells; AMPK activation; ↓ NOX4/COX-2; pro-apoptotic in irradiated cancer cells; antioxidant protection of normal tissues
EGCG + HPV E7 DNA vaccine *Green tea catechin—oral administration; immunoadjuvant in TC-1 syngeneic HPV+ model* [208]	Radiosensitisation	Preclinical/in vitro	Oral EGCG + E7/LAMP DNA vaccine → complete tumor regression in TC-1 HPV+ mouse model; ↑ E7-specific CD8+ T cells vs. vaccine alone; enhanced primary and memory T cell responses; no systemic toxicity, no hair loss, no lymphopenia—favorable safety profile for combination with RT/CT	↑ CD8+ cytotoxic T cell-mediated immunity; immunoadjuvant synergy with therapeutic HPV vaccine; no myelosuppression; NK cell and CTL potentiation
Resveratrol + cisplatin *Combination treatment—HNSCC cell line PE/CA-PJ49;* in vitro [242]	Chemosensitisation	Preclinical/in vitro	RSV + cisplatin: ↑ Tumor cell viability loss vs. cisplatin alone; ↑ p21 expression; ↑ apoptosis; ↓ cell cycle progression; selectivity for tumor cells over normal HUVEC endothelial cells; resveratrol amplifies cisplatin-induced DNA damage response via G1 arrest	↑ p21 (CDKN1A); G1 cell cycle arrest; ↑ apoptosis; amplifies cisplatin DNA double-strand break response; selectivity for tumour vs. normal cells
Curcumin + cisplatin *Combination treatment—HNSCC and HPV+ OPSCC cell lines;* in vitro [243]	Chemosensitisation	Preclinical/in vitro	Curcumin + cisplatin: ↑ Apoptosis and ↓ proliferation vs. monotherapy; ↓ IC50 of cisplatin when combined; ↑ p21; greater effect on tumor than normal cells. Curcumin targets CDC27 in HPV+ OPSCC cell lines (CU-OP-2/3/20); synergistic with PI3K inhibitor BYL719 and WEE1 inhibitor MK-1775	Synergistic apoptosis induction; ↓ cisplatin dose required for equivalent effect; p21↑; NF-κB inhibition; CDC27 targeting (HPV+ specific); PI3K/AKT/mTOR pathway
Honey + green propolis *Bee honey/Taiwanese green propolis—topical rinse and swallow; post-RT* [236]	Xerostomia/QoL	RCT	3-arm RCT (n = 75): Honey ↑ QoL scores (β = 10.05, *p* = 0.01) and ↓ OM severity; propolis ↓ patient-reported xerostomia vs. usual care; both superior to standard care across 12-week post-RT follow-up; well tolerated; GEE method analysis	↑ Salivary flow stimulation; antimicrobial (↓ Candida); mucosal hydration; anti-inflammatory (↓ salivary cytokines); flavonoid-mediated salivary gland protection
Coconut oil *Cocos nucifera—oil pulling/topical rinse; post-RT xerostomia management* [244]	Xerostomia/QoL	Clinical trial	Feasibility study (Ottawa HNC patients ≥18 months post-RT, ≥50 Gy): Coconut oil safe, inexpensive, well tolerated; patient-reported ↓ dry mouth symptoms; no adverse events; identified as promising low-cost intervention; larger RCT required for efficacy confirmation	Mucosal lubrication (lauric acid and medium-chain fatty acids); antimicrobial (lauric acid); anti-inflammatory; ↓ friction and mucosal dryness symptoms
Aloe vera *Aloe barbadensis—gel 3× daily; post-RT xerostomia and mucosal healing* [239]	Xerostomia/QoL	Clinical trial	Active RCT NCT06381635 (Ain Shams University, 2024): Testing aloe vera gel + Manuka honey for RT-induced mucositis and xerostomia in HNC patients; salivary TGF-β1 and EGF as biomarkers; prior studies confirm aloe vera ↓ burning sensation, improves mucosal hydration and epithelial healing post-RT	Acemannan polysaccharides: ↑ Fibroblast proliferation + epithelial repair; ↑ EGF; ↓ IL-1β; humectant (↓ water loss from mucosa); antimicrobial

↓—inhibition; ↑—activation.

**Table 6:** Phytochemicals and their antiviral potential on HPV-induced papillomatosis.

Constituent/Formulation	Anatomical Site	HPV Types Targeted	Evidence Level	Year	Key Findings	OPSCC Preventive Link	Mechanism of Action
Sinecatechins (Veregen^®^) *Polyphenon E—EGCG-rich green tea extract; 15% topical ointment 3× daily for up to 16 weeks* [245]	Genital/perianal warts	HPV 6, HPV 11 (low-risk); active against HPV 16 in vitro	FDA-approved/RCT/Phase II–III	2006/2009	FDA-approved botanical drug (2006, first ever) for condylomata acuminata. Phase III RCTs (n = 1005): 15% ointment → 52–57% wart clearance vs. 33–37% placebo; lower recurrence rate vs. imiquimod; active on oral and anal warts. Full wart clearance is maintained with fewer relapses than podophyllotoxin.	Direct antiviral/prevention	↓ AP-1 transcription → ↓ HPV gene expression; EGCG ↓ HPV E6/E7; pro-apoptotic; anti-inflammatory; immunostimulatory (↑ CD8+ T cell response); antiproliferative
Sinecatechins—post-ablation sequential therapy *10% ointment after CO2 laser or cryotherapy; PACT-II trial* [223]	Genital/perianal warts	HPV 6, HPV 11	RCT/Phase II–III	2022	PACT-II prospective assessor-blinded pilot RCT: Sinecatechins 10% applied after cryotherapy ablation of external genital warts → significantly ↓ reduced condyloma recurrence rate vs. no further treatment; well tolerated; supports sequential strategy combining rapid ablative clearance with botanical immunostimulation to prevent viral re-establishment.	Indirect/mechanistic	↓ Viral reservoir post-ablation; EGCG immunostimulation ↑ local HPV-specific immunity; ↓ risk of viral spread to oropharynx via reduced genital load
Polyphenon E/EGCG (oral) *Green tea catechin extract—200 mg oral capsule daily; 8–12 weeks* [219]	Cervico-vaginal	HPV 16, HPV 18 (high-risk); persistent infection	RCT/Phase II–III	2003/2014	Ahn et al.: 69% clinical response in HPV-infected cervical lesions vs. 10% untreated controls (n = 51). Garcia et al. Phase 2 RCT: 17.1% complete response (HPV DNA negative + normal histology) with Polyphenon E in persistent high-risk HPV + CIN1. Oral + topical combined approach: 74–75% response rate; EGCG capsule alone: 60% response.	Direct antiviral/prevention	↓ AP-1 → ↓ HPV transcription; EGCG ↓ E6/E7 oncoprotein levels; ↑ p53; immunostimulatory; ↓ viral persistence; epigenetic (DNMT inhibition); ↓ cell proliferation
EGCG + Folic acid + Vitamin B12 + Hyaluronic acid (Pervistop^®^) *Oral supplement: 200 mg EGCG + 400 mcg folic acid + 1 mg B12 + 50 mg HA; 1–2 caps/day for 3–6 months* [246]	Cervico-vaginal + Anal	Multiple high-risk HPV types (cervical + anal)	Clinical trial/study	2023/2024	Calcagno et al. 2024 [246]: 5 clinical cases with persistent cervical or anal HPV infection—all 5 patients had HPV DNA negative and cytological lesions improved after 3–6 months of Pervistop^®^. Prior clinical study (2023) confirmed HPV clearance and CIN regression with this combination. In vitro: EGCG + FA + B12 + HA combination synergistically ↑ induces apoptosis, ↑ p53, ↓ E6/E7 (stronger than any single molecule).	Direct antiviral/prevention	EGCG: ↓ E6/E7, ↑ p53; Folic acid: ↑ DNA integrity, ↓ viral integration risk; B12: ↑ immune surveillance; HA: mucosal integrity; synergistic antiviral + immunosupportive
Myrtle (*Myrtus communis*) *Vaginal suppository: 10% aqueous extract + 0.5% essential oil; 20 suppositories per menstrual cycle × 3 months* [247]	Cervicovaginal	HPV 16, HPV 18, and other high-risk cervicovaginal types	RCT/Phase II–III	2018	Nikakhtar et al. RCT (n = 60 women with cervicovaginal HPV): HPV DNA negative at end of study 92.6% vs. 62.6% placebo (*p* = 0.036); cervical lesion size reduction 71.4% vs. 30.4% (*p* = 0.015). Separate clinical study: topical myrtle leaf poultice superior to salicylic acid for hand/foot warts. 2025 in silico study (virtual screening): Active phytochemicals dock to HPV L1 capsid protein, E6, and E7 targets.	Direct antiviral/prevention	Tannins and flavonoids (myricetin, quercetin, ellagic acid): antiviral (↓ HPV replication), antimutagenic, pro-apoptotic; ↓ viral persistence; L1 capsid binding confirmed in silico
Curcumin + polyherbal vaginal cream (Basant) *Curcumin + reetha + amla (Phyllanthus emblica) + aloe vera—vaginal cream 1 application/day × 30 days* [248]	Cervicovaginal	Multiple high-risk HPV types	RCT/Phase II–III	2013	Basu et al. Phase II RCT (n = 287 HPV+ women): Curcumin vaginal capsules and Basant polyherbal cream → HPV clearance significantly greater than placebo arms; amla + reetha + curcumin combination showed non-significant trend toward greater HPV 16/18 clearance; well tolerated (mild vaginal irritation in Basant arm only); supports curcumin as anti-HPV topical agent.	Indirect/mechanistic	Curcumin: ↓ HPV E6/E7, ↑ p53, ↓ NF-κB; Amla (ellagic acid, vitamin C): antiviral, ↑ immunity; Aloe vera: ↑ mucosal immunity (acemannan); Reetha: antimicrobial + antiviral saponins
Curcumin (oral/intravaginal—ongoing RCTs) *Oral 500 mg twice daily for CIN3 (Baylor RCT) OR intravaginal 2000 mg weekly × 20 weeks (Emory RCT)* [249]	Cervicovaginal + Oral/OPSCC prevention	HPV 16, HPV 18 (CIN 2/3, high-risk)	RCT/Phase II–III	2020/ongoing	Baylor RCT (NCT02554344): Oral curcumin 500 mg twice daily × 12 weeks for CIN3—colposcopy and NF-κB assessed at 6 and 12 weeks. Emory study: Intravaginal curcumin for LSIL/HSIL in women with and without HIV. Curcumin nanoemulsion (2025): ↓ HPV E6/E7 oncogenes in TC-1 HPV+ model in vitro and in vivo. Systematic review (2024): A meta-analysis of curcumin supports CIN regression and HPV clearance.	Direct antiviral/prevention	↓ HPV E6/E7 → ↑ p53/Rb restoration; ↓ NF-κB; ↓ AP-1; ↓ CIN progression; curcumin nanoemulsion ↑ bioavailability and intratumoural delivery
Herbal treatments—systematic review *Polyphenon E (oral), curcumin vaginal, myrtle vaginal, EGCG oral, olive oil vaginal spray* [250]	Cervicovaginal	Multiple HPV types (high-risk and low-risk)	Systematic review	2024	Demir & Cetinkaya systematic review (RCTs up to December 2023): Herbal treatments show a significant reduction in ASCUS/LSIL cytological abnormalities; moderate evidence for HPV DNA clearance; most robust evidence for Polyphenon E (oral), myrtle suppository, and curcumin vaginal capsules; all well tolerated; authors conclude herbal interventions represent a promising and safe strategy for HPV clearance.	Indirect/mechanistic	Class-level antiviral + immunostimulatory effect across polyphenols, terpenes, and essential oils; systematic clearance of high-risk HPV from cervicovaginal epithelium = primary prevention for HPV-driven cancers, including OPSCC
Indole-3-carbinol (I3C) *Cruciferous vegetables (broccoli, cabbage, cauliflower)—oral 200–400 mg adults twice daily; weight-based in children* [251,252]	Respiratory (RRP)	HPV 6, HPV 11 (RRP); HPV 16/18 (CIN)	Clinical trial/study	1998/2004	Derkay et al. Phase I (1998): Established the safety and tolerability of I3C for RRP in adult and pediatric patients. Rosen & Bryson long-term clinical study (2004, n = 45 RRP patients, 5-year follow-up): 11/33 (33%) experienced remission and required no further surgery while on I3C; no immediate or long-term side effects; pediatric response confirmed. Separate preliminary trial: I3C 200–400 mg/day resulted in a statistically significant regression of CIN 2/3. Listed in the University of Iowa RRP treatment protocol (updated January 2023).	Direct antiviral/prevention	↑ 2-OHE1:16α-OHE1 ratio (anti-oestrogenic); ↓ NF-κB; ↓ PI3K/AKT; ↓ cyclin D1; ↓ Wnt/β-catenin; ↓ HPV-driven epithelial proliferation; ↓ malignant transformation risk of RRP
Diindolylmethane (DIM) *Acid-catalyzed dimeric metabolite of I3C—oral supplement 108–300 mg/day* [183]	Respiratory (RRP)	HPV 6, HPV 11	Preclinical + clinical	Ongoing	DIM is the stable bioactive metabolite of I3C formed during digestion; it shares I3C’s anti-HPV properties but with superior bioavailability and chemical stability. Used clinically as an adjunct to surgery in RRP management. DIM 108 mg/day (30-day clinical trial): ↑ Urinary 2-OHE1 in post-menopausal women. DIM 300 mg/day (thyroid cancer trial): Modified estrogen metabolism confirming systemic bioactivity. Preclinical: ↑ Immune surveillance in HPV+ cells; ↓ papilloma recurrence in model systems.	Indirect/mechanistic	↑ 2-OHE1:16α-OHE1 ratio; ↓ NF-κB; ↓ Wnt/β-catenin; ↑ p27/p21; immunomodulatory; ↓ HPV-driven respiratory epithelium proliferation; ↓ malignant transformation risk in RRP
*Nigella sativa + Moringa oleifera* + *Musa acuminata* peel *Herbal mixture; polymer film-forming topical system for plantar/cutaneous HPV warts* [253]	Multi-site/plantar	HPV 1, 2, 4 (cutaneous); network pharmacology targets HPV 6/11/16/18	RCT/Phase II–III	2024	Polymer film herbal mixture significantly ↓ reduced wart size and quantity vs. placebo in outpatient clinic patients. LC-HRMS identified 49 bioactive metabolites. Network pharmacology: Thymoquinone (N. sativa), isothiocyanates (Moringa), and lectins (Musa) target HPV-relevant genes, including EGFR, TP53, and AKT1. Thymoquinone is antiviral in separate studies, including ↓ a reduction in HPV E6 expression.	Preclinical pathway	Thymoquinone (N. sativa): ↓ NF-κB, ↓ PI3K/AKT, antiviral; Isothiocyanates (Moringa): pro-apoptotic, ↑ p53; Lectins (Musa): immune stimulation; ↓ HPV viral load in infected epithelium

↓—inhibition; ↑—activation.

## Data Availability

No new data were created or analyzed in this study. Data sharing is not applicable to this article.

## Supplementary Materials

The following supporting information can be downloaded at https://www.mdpi.com/article/10.3390/cimb48060626/s1. Table S1: Comparative Evidence of HPV-HNSCCs: OPSCC vs. OSCC vs. LSCC.

## Abbreviations

The following abbreviations were used in the manuscript:

^13^C3	carbon-13 labeled compound with 3 atoms of ^13^C
2D	two-dimensional
2R,3R	the spatial orientation of two chiral centers in a molecule
93VU147T	research compound identifier
AJCC	American Joint Committee on Cancer
AKT	protein kinase B
AP-1	activator protein 1
ARE	antioxidant response element
B1	regulatory protein
Bax	B-cell lymphoma 2-associated X protein
Bcl-2	B-cell lymphoma 2
Bcl-xL	B-cell lymphoma-extra large
BIM	Bcl-2 Interacting Mediator of Cell Death
C/EBPβ	CCAAT/Enhancer-Binding Protein beta
CCAAT	short DNA sequence
C57BL/6	cell line, a productive inbred strain of lab mouse
Ca9-22	cell-line human gingival squamous carcinoma-derived cells
CaSki	human cervical cancer cell line
CBP	CREB-binding protein
CD8+	cluster of differentiation 8–positive
CDK	cyclin-dependent kinase
CDK1-6	cyclin-dependent kinases 1- 6
CDKN1A	cyclin-dependent kinase inhibitor 1
c-Ha-ras	cellular Harvey rat sarcoma viral oncogene homolog
CI	confidence interval
cIAP-2	cellular inhibitor of apoptosis protein 2
c-Myc	cellular myelocytomatosis oncogene
COX-2	cyclooxygenase-2
CR	clinical response
CREB	cAMP response element-binding protein
CRT	chemoradiotherapy
Cyclin D1	cyclin family protein
DIM	diindolylmethane
DNA	Deoxyribonucleic Acid
DNMT	DNA methyltransferase
DNMT1	DNA (cytosine-5)-methyltransferase 1
DR5	death receptor 5
DREAM	DP, RB-like, E2F, and MuvB complex—protein complex
DP	dimerization partner protein
RB-like	retinoblastoma-like
E2F	E2 promoter-binding factors
MuvB	multi-vulval class B
E1A	early region 1A (adenoviral protein)
E2F1-5	E2 promoter-binding factors 1–5
E6/E7	HPV early proteins 6 and 7
E6-AP	E6-associated protein
E6–E6AP	viral protein E6 and cellular protein E6AP
ECE	extranodal extension/extracapsular
ECG	epicatechin gallate
ECOG-ACRIN 3311	clinical study
ECOG	Eastern Cooperative Oncology Group
ACRIN	American College of Radiology Imaging Network
3311	the study number in the internal registry
EGCG	epigallocatechin -3-gallate
EGFR	epidermal growth factor receptor
EMT	epithelial–mesenchymal transition
ENE	extranodal extension
ERK	extracellular signal-regulated kinase
ERK1/2	extracellular signal-regulated kinases 1 and 2
FADD	fas-associated protein with death domain
FAS	fas cell surface death receptor
Fas/FasL	receptor–ligand system involved in apoptosis induction
FDA	Food and Drug Administration
Fe^2+^	the divalent iron ion
G1/S	gap 1 synthesis
G2/M	“gap2” face to the mitosis phase
GJIC	gap junctional intercellular communication
GPX4	glutathione peroxidase 4
GSK3β	glycogen synthase kinase 3 beta
Gy	gray
H&N EXAM	head and neck examination
HaCaT	human keratinocyte cell line
HDAC	histone deacetylase
HeLa	human cervical cancer cell line
HES1	hairy and enhancer of split 1
HEY1	hairy/enhancer-of-split related with YRPW motif 1
HIF-1α	hypoxia-inducible factor 1 alpha
HNSCC	head and neck squamous cell carcinoma
HO-1	heme oxygenase 1
HPV	human Papillomavirus
HPV 16	human Papillomavirus 16
HPV 18	human Papillomavirus 18
HPV E6	human Papillomavirus early protein 6
HPV L1	human Papillomavirus L1 protein
HPV OPC	human Papillomavirus–positive oropharyngeal cancer
HPV PCR	human Papillomavirus polymerase chain reaction
HPV URR	human Papillomavirus upstream regulatory region
HPV+	human Papillomavirus positive
HPV16-E6	human Papillomavirus 16 Early protein 6
HPV16+ TC-1	HPV16 positive TC-1 cell line
HPV68	human Papillomavirus type 68
HSC-3	cell-line, cells derived from human oral squamous carcinoma
HSC-4	cell-line, cells derived from human oral cavity squamous cell carcinoma
hTERT	human TERT
TERT	telomerase reverse transcriptase
I3C	indole-3-carbinol
IHC	immunohistochemistry
IL-1β	interleukin-1 beta
IL-6	interleukin 6
IL-8	interleukin 8
IMRT	intensity-modulated radiation therapy
ITCs	isothiocyanates
JAK	janus kinase
KCNH1	a voltage-gated potassium channel
KEAP1	kelch-like ECH-associated protein 1
ECH	Echinoderm microtubule-associated protein
LNPs	lipid nanoparticles
LXCXE	leucine–any amino acid–cysteine–any amino acid–glutamic acid
MAPK	mitogen-activated protein kinase
CH	cysteine/histidine
MARCHF8	membrane-associated RING-CH-type finger 8
RING	really interesting new gene
MDM2	mouse double minute 2
MDSCs	myeloid-derived suppressor cells
MHC-1	major histocompatibility complex class I
miR-31	microRNA-31
miRNA	microRNA
MMP-2	matrix metalloproteinase-2
MMP-9	matrix metalloproteinase-9
MRI	magnetic resonance imaging
mRNA	messenger RNA
mTOR	mechanistic target of rapamycin
MTT	methyl/thiazolyl/tetrazolium
MYC	myelocytomatosis oncogene
NF-κB	nuclear factor kappa-light-chain-enhancer of activated B cells
nHOK	normal human oral keratinocytes
NK	natural killer
NOTCH1	neurogenic locus notch homolog protein 1
NQO1	quinone/oxidoreductase/the first identified member of the family
Nrf2	nuclear factor erythroid 2-related factor 2
HNSCC	head & neck squamous cell carcinoma
OPCs	oligodendrocyte progenitor cells
OPSCC	oropharyngeal squamous cell carcinoma
OSCC	oral squamous cell carcinoma
p16	protein 16
p16^INK4a	inhibitor of cyclin-dependent kinase 4a
p21	tumor suppressor protein
p27	protein 27
p300	E1A-binding protein p300
p53	tumor protein p53
PARP	Poly (ADP-ribose) polymerase
ADP	adenosine diphosphate
PATHOS	post-operative adjuvant treatment for HPV-positive tumors
PD-1	programmed cell death protein 1
PD-L1	programmed death-ligand 1
pEGFR	phosphorylated epidermal growth factor receptor
PET-CT	positron emission tomography–computed tomography
pH	potentia hydrogenii
PI3K	phosphoinositide 3-kinase
PLGA	poly-lactic-co-glycolic acid
PORT	post-operative radiotherapy
pRb	retinoblastoma protein
pSTAT3	phosphorylated STAT3
pT	pathological tumor stage
PTEN	phosphatase and TENsin homolog
PUMA	p53 upregulated modulator of apoptosis
RAS	rat sarcoma
Rb	retinoblastoma protein
RCT	randomized controlled trial
ROS	reactive oxygen species
RT	radiotherapy
SCC	squamous cell carcinoma
SCC25	cell line—human oral squamous carcinoma-derived cells
SOX2	SRY-Box Transcription Factor 2
SRY	sex-determining region Y
S-phase	synthesis phase
STAT	signal transducer and activator of transcription
STAT3	signal transducer and activator of transcription 3
STUB1	STIP1 homology and U-box containing protein 1
STIP	stress-induced phosphoprotein 1
T cells (Tregs)	regulatory T lymphocytes
T3	primary tumor
T4	locally advanced tumor
TC-1	murine (mouse) tumor cell line
TGF-β1	transforming growth factor beta 1
TLM	transoral laser microsurgery
TME	tumor microenvironment
TNF-α	tumor necrosis factor alpha
TNM	tumor/nodes/metastasis
TORS	transoral robotic surgery
TP53	tumor protein 53
TNF	tumor necrosis factor
TRAIL	TNF-related apoptosis-inducing ligand
TriCurin	curcumin/epicatechin gallate/resveratrol
TSH	thyroid-stimulating hormone
URR	upstream regulatory region
uVIN	usual vulvar intraepithelial neoplasia
VEGF	vascular endothelial growth factor
WFA	wisteria floribunda agglutinin
Wnt/β-catenin	wingless/integrated β-catenin = beta-catenin
XIAP	X-linked inhibitor of apoptosis protein
Y705	tyrosine (Y) at position 705
YRPW	tyrosine/arginine/proline/tryptophan—short amino acid motif
α-MG	α-methylglucoside
β-catenin	beta-catenin
μM	micromolar
