# Supplementary material for: Plant Bioactive Constituents and Their Potential Benefits in HPV-Positive Oropharyngeal Squamous Cell Carcinoma—A Narrative Review
Source: Curr Issues Mol Biol. 2026 Jun 16;48(6):626. doi: 10.3390/cimb48060626 (PMC13297950; doi:10.3390/cimb48060626)
Supplement: Supplementary file 1 [file cimb-48-00626-s001.zip › cimb-4347313-supplementary.pdf]

Table S1. Comparative Evidence of HPV-HNSCCs: OPSCC vs. OSCC vs. LSCC

Head and Neck Squamous Cell Carcinomas – Oropharyngeal, Oral Cavity, and Laryngeal Subtypes

✓ Established / Favorable ~ Controversial / Unclear ✗ Not established / Adverse — Not applicable

Parameter	HPV-OPSCC *Oropharyngeal SCC*	HPV-OSCC *Oral Cavity SCC*	HPV-LSCC *Laryngeal SCC*
EPIDEMIOLOGY & PREVALENCE			
**Primary anatomical subsites**	Palatine tonsils, base of tongue (BOT), soft palate, posterior oropharyngeal wall	Oral tongue (ant. 2/3), floor of mouth, buccal mucosa, hard palate, alveolar ridge, lip	Supraglottis (~27%), glottis (~70%), subglottis (~3%); p16+ associated with supraglottic location
**Global HPV prevalence**	42% (95% CI 36–49%)<br>Up to 40–90% in developed nations; 56% in North America	10% (95% CI 7–13%)<br>3.6–22% by HPV DNA testing; wide geographic variation	20–30% HPV DNA positivity<br>True causal prevalence estimated <10%; bystander common
**Causal role of HPV**	✓ Firmly established – IARC Group 1 carcinogen	~ Controversial – Often bystander; multifactorial etiology	✗ Limited / uncertain – Often bystander; tobacco remains dominant driver
**Dominant HPV genotype**	HPV-16 (>90% of HPV+ cases)<br>Consistently active E6/E7; high viral load; genome integrated	HPV-16 (69% of HPV+ OSCC)<br>HPV-18 also present; mixed-type infections common	HPV-16 dominant, followed by HPV-18<br>E6/E7 mRNA often undetectable despite HPV DNA positivity
**Global incidence trend**	✗ Increasing<br>AAPC +3.76%/yr; tonsil +4.63%/yr; base of tongue +4.79%/yr	~ Stable / context-dependent<br>Oral tongue SCC rising since 1999 (APC +2.36%); HPV+ subset trend unclear	✓ Declining<br>AAPC −2.56%/yr driven by reduced tobacco exposure
PATIENT PROFILE & RISK FACTORS			
**Typical demographics**	Younger (40–60 yrs), male, white, non-smoker; HPV acquired sexually	Older; predominantly tobacco/alcohol/betel nut users; no HPV-specific demographic shift	Predominantly tobacco (88%) and alcohol (56%) users; no HPV-driven demographic change
**Primary risk factors**	Oral HPV infection (sexually transmitted)<br>Tobacco/alcohol secondary in HPV+ subset	Tobacco, alcohol, betel nut (75–80% of cases)<br>HPV minor / secondary role; causal pathway unclear	Tobacco (dominant carcinogen), alcohol<br>HPV role unproven as primary driver
MOLECULAR BIOLOGY & DIAGNOSTICS			
**p16 IHC as HPV surrogate**	✓ Validated – AJCC 8th preferred assay<br>≥70% nuclear + cytoplasmic cutoff; highly specific in oropharynx; gold standard for clinical staging	✗ Not validated – p16+++ only 0.7% of OSCC<br>Unreliable correlation with HPV DNA; non-viral mechanisms drive p16 expression in oral cavity	✗ Not reliable – p16+ in ~7.9% of LSCC only<br>Poor kappa agreement with HPV DNA; p16 not prognostic in larynx; other non-viral p16 drivers exist
**Gold-standard diagnostic test**	p16 IHC alone (clinical standard)<br>HPV DNA PCR + E6/E7 mRNA for research/equivocal cases	HPV DNA PCR + E6/E7 mRNA co-detection required<br>p16 alone insufficient for causal determination	HPV DNA PCR + E6/E7 mRNA co-detection required

Parameter	HPV-OPSCC<br>*Oropharyngeal SCC*	HPV-OSCC<br>*Oral Cavity SCC*	HPV-LSCC<br>*Laryngeal SCC*
			HPV DNA alone is insufficient; bystander infection is common in the larynx
**E6/E7 transcriptional activity**	✓ **Consistently active** – Integrated; high viral load; robust oncoproteins	~ **Often absent** – HPV DNA present without active E6/E7 transcription	✗ **Rarely detectable** – HPV RNA undetectable in surgical series despite DNA+
**TP53 mutation pattern**	Rare (<10%)<br>E6-mediated proteasomal p53 degradation; no somatic mutation needed	Frequent (>50%)<br>Dominant carcinogenic mechanism: tobacco-related TP53 hotspot mutations	Frequent<br>Tobacco/carcinogen-driven; supraglottis has the highest smoking-associated SNV burden (TCGA)
**Key co-occurring molecular alterations**	PIK3CA mutations (~50%)<br>Wnt/β-catenin activation; low overall somatic mutation burden; CDKN2A usually intact	TP53, CDKN2A, CASP8, FGFR2/3 mutations<br>High somatic mutation burden; EGFR aberrations are rare in the HPV+ subset	TP53, CDKN2A, EGFR overexpression, cyclin D1 amplification<br>Supraglottis highest mutational load; PI3K/AKT/mTOR differentially enriched
**Routine HPV testing recommended**	✓ **Yes — mandatory** – All newly diagnosed OPSCC; guides staging and treatment planning	✗ **No** – Not currently recommended in clinical guidelines	✗ **No** – Results do not alter management; not standard of care
PROGNOSIS & CLINICAL OUTCOMES			
**Effect of HPV+ on prognosis**	✓ **Strongly favorable** – Independent positive prognostic factor<br>HPV− has 4.5× higher hazard of death in multivariable analysis	✗ **Adverse/neutral** – Meta-analysis: decreased OS and disease control<br>HPV infection in OSCC is an adverse prognostic factor (Laryngoscope 2022, PMID 34953144)	~ **Inconclusive** – No significant OS difference by HPV status<br>Not an independent prognostic factor in multivariable analyses; conflicting reports
**5-yr OS / DFS – HPV+ vs HPV−**	HPV+: >80% DFS; median OS ~130 months; 2-yr OS 90.9%<br>HPV−: <50% DFS; 2-yr OS 73.6% ($p<0.001$)	HPV+: no significant survival benefit; possible worsening (HR >1 in meta-analysis)<br>HPV−: comparable or better OS	HPV+ DNA: OS not significantly different from HPV− (log-rank $p = 0.64$)<br>HPV mRNA: insufficient data for reliable comparison
**Enhanced radiosensitivity**	✓ **Well established** – E6/E7-mediated cell cycle deregulation, impaired DNA repair, and immune activation post-RT	✗ **Not established**	✗ **Not established**
**Recurrence pattern**	HPV+ recurs earlier but less frequently<br>HPV− shows later, more persistent recurrence	No established HPV-specific recurrence difference	No established HPV-specific recurrence pattern
**Tumor microenvironment**	Rich immune infiltration; HPV-specific CD8+ T-cells present<br>PD-1/PD-L1 expression; immune-rich (IR) TME subtype exclusively HPV+	Tobacco-dominant TME; immune infiltration not HPV-driven<br>No HPV-specific immune phenotype; sparse comparative data	Tobacco-shaped TME; no established HPV-specific immune phenotype<br>EGFR overexpression dominant; distinct from OPSCC immune profile
STAGING & TREATMENT			

Parameter	**HPV-OPSCC**<br>*Oropharyngeal SCC*	**HPV-OSCC**<br>*Oral Cavity SCC*	**HPV-LSCC**<br>*Laryngeal SCC*
**Separate AJCC staging system**	**✓ Yes — AJCC 8th (2017)** – Separate cTNM + pTNM; Stage IV reserved for M1 only<br>AJCC 9th refinement currently in validation (Lancet Oncol. 2025)	**✗ No** – Standard HNSCC TNM; HPV status does not modify staging	**✗ No** – Standard TNM; no HPV-based staging modification in current guidelines
**De-escalation strategies**	**✓ Active clinical trials** – NRG HN002, PATHOS, OPTIMA<br>Reduced RT dose (50–60 Gy) and/or cisplatin substitution under evaluation; basis: favorable biology + radiosensitivity	— Not applicable<br>Standard-dose treatment; no trials currently based on HPV status alone	— Not applicable<br>Standard protocols regardless of HPV; no de-escalation rationale
**Standard treatment**	CRT (70 Gy + cisplatin); TORS for T1–T2<br>Pembrolizumab/nivolumab for recurrent or metastatic disease	Surgery ± adjuvant RT/CRT<br>No HPV-based treatment modification in current guidelines	Organ-preservation CRT (advanced); TLM / partial laryngectomy (early)<br>No HPV-based modification; prognosis driven by subsite, stage, and tobacco status
